# Regional Personality Differences in Great Britain

**DOI:** 10.1371/journal.pone.0122245

**Published:** 2015-03-24

**Authors:** Peter J. Rentfrow, Markus Jokela, Michael E. Lamb

**Affiliations:** 1 Department of Psychology, University of Cambridge, Cambridge United Kingdom; 2 Institute of Behavioural Sciences, University of Helsinki, Helsinki, Finland; Georgia State University, UNITED STATES

## Abstract

Recent investigations indicate that personality traits are unevenly distributed geographically, with some traits being more prevalent in certain places than in others. The geographical distributions of personality traits are associated with a range of important political, economic, social, and health outcomes. The majority of research on this subject has focused on the geographical distributions and macro-level correlates of personality across nations or regions of the United States. The aim of the present investigation was to replicate and extend that past work by examining regional personality differences in Great Britain. Using a sample of nearly 400,000 British residents, we mapped the geographical distributions of the Big Five Personality traits across 380 Local Authority Districts and examined the associations with important political, economic, social, and health outcomes. The results revealed distinct geographical clusters, with neighboring regions displaying similar personality characteristics, and robust associations with the macro-level outcome variables. Overall, the patterns of results were similar to findings from past research.

## Introduction

Geography is important for understanding a variety of behavioral outcomes. For instance, research in regional economics examines the ways in which local resources, infrastructure, and amenities influence residential mobility and happiness. Research in public health investigates the impact that social and economic factors have on health and well-being. And research in certain areas of political science focus on the ways in which demographic, social, and economic factors shape public opinion and affect election returns. A common theme to emerge from all of that research is that there are strong links between the locations in which people live and their attitudes, motivations, and well-being—constructs that are central to psychology. And yet, psychological scientists have only recently recognized the relevance of geography for understanding such important and widely studied phenomena.

Indeed, the past few years have witnessed growing interest in geographical psychology—the study of the spatial organization of psychological phenomena and the impact that individual characteristics, social institutions, and the environment have on that organization [1]. The most prominent strands of research focus on the geographical distribution of psychological characteristics [2–5], the links between geographical psychological characteristics and political, economic, social and health (PESH) indicators [6–8], the psychological basis of residential mobility [9–13], and the impact of the physical environment on attitudes, beliefs, and emotions [14–20].

The present research falls squarely within the first two research strands and is concerned with the geographical distribution of personality in Great Britain and its associations with PESH indicators. To date, most of the research concerned with geographical differences in personality and their PESH correlates has focused on variation across nations, and only a handful of studies have examined variation within nations, nearly all of which have focused on the United States. Thus, the current project was designed to replicate and extend research in this burgeoning area by assessing the degree to which geographical variation in personality occurs in other countries and whether similar patterns of associations between the geographical personality traits and PESH indicators emerge.

### Geographical Variation in Personality

The idea that there are geographical differences in personality stems from three key lines of research. The first focuses on social influence. The basic idea is that the traditions, customs, lifestyles, and daily practices common to an area affect social norms, which in turn affect people’s attitudes and behaviors. This is the assumption underlying many studies of cross-national psychological differences [21–22]. The second line of research focuses on ecological influence, or the idea that features of the physical environment affect people’s thoughts, feelings, and behaviors. One influential program of research suggests that geographical differences stem from the historical prevalence of infectious disease, with unhealthy environments encouraging more cautious and risk-averse traits [14, 15, 17]. There is also evidence that climates and economic conditions interact to affect people’s values and beliefs [19, 20]. The third line of research focuses on selective migration, or the hypothesis that people migrate to places that satisfy and reinforce their psychological needs. Recent research indicates that people who are creative and sociable are more likely to migrate than are people low on those traits [9,10], and that people who are agreeable are less inclined to move from their hometowns than people who are less friendly [23]. This work suggests that geographical differences in personality could emerge as a result of genetic drift.

Research on social influence, ecological influence, and genetic influence provide compelling empirically based reasons to expect geographical variation in various psychological characteristics. But what evidence is there for such variation? Most of the research on geographical variation in psychological characteristics has focused on cross-national differences, with a few studies focusing on regional variation. All of the studies have focused on variation in either personality or well being, with both lines of work revealing robust differences across and within countries.

Interest in the geographical distribution of personality stems primarily from the establishment of the Big Five framework (i.e., Extraversion, Agreeableness, Conscientiousness, Neuroticism, Openness) as an empirically based and widely accepted model for conceptualizing the structure of personality [24, 25]. Analyses of national personality differences and their links with PESH indicators are usually based on the mean personality scores derived from a sample of respondents in each nation. So to say, for example, that Switzerland is high in Conscientiousness is to say that the mean level of Conscientiousness derived from a sample of Swiss residents is high compared to the mean levels of Conscientiousness derived from samples of residents of other countries. Systematic comparisons of nation-level mean personality scores reveal considerable variability in each of the Big Five personality domains [7, 26, 27]. For example, citizens of Asian countries score lower on measures of Extraversion than citizens of other cultures; citizens of Central and South American nations score higher on measures of Openness; and citizens of Southern and Eastern European nations score higher in Neuroticism than do residents of Western European countries [2, 28]. Moreover, analyses of the PESH correlates of national-personality scores have revealed associations between national levels of Neuroticism, for example, and rates of cancer, smoking, obesity, and life expectancy [7], suggesting that the prevalence of anxious and depressed individuals is associated with poor health practices and disease prevalence at a national level.

Although considerable attention has been given to cross-national comparisons of personality, less attention has been paid to the ways in which these characteristics vary within countries. Research in geographical sciences has shown regional variation on a number of indicators—including public opinion, economic prosperity, crime, and morbidity—that have links to personality, so it seems reasonable to expect regions to vary on particular personality traits, too.

Indeed, evidence from a few large-scale investigations has revealed reliable variance in personality across the U.S. and one study has revealed variance across regions of the Russian Federation. Four investigations have explored the geographical distribution of personality across large multi-state regions and states [3, 4, 29, 30], the results from which have converged to indicate that there are robust geographical patterns in the distributions of personality traits across the U.S. For example, residents of the New England, Middle-Atlantic, and Pacific regions generally score higher in Openness than residents of the Great Plains, Midwest, and Southeast; residents of the South score higher in Agreeableness than people living in the Northeast; and residents of the Northeast and Southeast score higher in Neuroticism than do residents of the Midwest and West. Furthermore, these regional differences in personality have been linked to several important PESH outcomes. For example, state-level Openness is related to liberal public opinion, human capital, and economic prosperity [4, 8, 31, 32]; Agreeableness is linked to economic equality, social capital, and low crime [6, 33]; and Neuroticism is related to various indicators of poor health [4, 34–36]. There is also evidence for regional personality differences across the Russian Federation, which showed links between high Openness and economic prosperity [37]. The associations between aggregate-level personality traits and PESH outcomes indicate that the psychological characteristics that are prevalent in a region are associated with a range of important macro-level indicators, from voting patterns and academic achievement to crime and mortality.

Much of the research on geographical personality differences has focused on the prevalence of individuals with specific psychological characteristics and how the prevalence of those characteristics relate to PESH indicators. The research is useful because it provides information about how places compare on particular psychological traits and it reveals the degree to which psychological processes generalize across multiple levels of analysis and cultures. However, nearly all of the studies of regional personality differences have been carried out in the U.S. Consequently, it is unclear whether regional variations in the same psychological characteristics are evident in other countries, or whether similar patterns of associations emerge between regional psychological and PESH indicators. Extending research on regional differences to other countries has the potential to inform our understanding of the ways in which certain psychological traits cluster together geographically and become expressed on important macro-level indicators.

### The Present Investigation

The aim of the present investigation was to examine regional variation in personality and its links with important PESH outcomes in Great Britain. Specifically, the present investigation asked: Is there reliable variance in personality across regions of the country? How are the Big Five personality traits geographically distributed? And how do the aggregate personality traits relate to the PESH characteristics of regions?

To address our research questions, we examined regional variation across Local Authority Districts (LADs) in Great Britain. In England, LADs correspond to London boroughs, metropolitan districts, unitary authorities, and non-metropolitan districts, in Wales they correspond to unitary authorities, and in Scotland they correspond to council areas. The current study used the 2009 LAD boundaries, which included 380 LADs. It is important to emphasize that, compared to previous analyses of regional psychological differences at the state or national levels, the current project focused on geographical regions that are significantly smaller in area and population. As a result, this project allows for a more fine-grained analysis of regional variation that can reveal differences between urban and rural environments and even differences within large cities.

#### Predictions

The main goals of this investigation were to explore how the Big Five personality traits were geographically distributed in Great Britain and how they related to important PESH indicators. We made no specific predictions about the precise geographical distribution of the personality traits. However, results from previous studies on geographical personality clustering indicate that physical proximity is related to personality similarity [2, 3, 28], so we expected neighboring LADs to be psychologically similar.

Our expectations about how the LAD-level personality scores would relate to the PESH indicators were informed by past research in the U.S. [3–5, 32–36, 38]. Extraversion reflects individual differences in energy and sociability, but no consistent patterns of PESH relations have emerged for Extraversion at national or state levels, so we made no explicit predictions about how LAD-level Extraversion would relate to the PESH indicators. Agreeableness reflects individual differences in prosocial behavior and research in the U.S. suggests that aggregate-level Agreeableness is positively related to social capital and negatively related to rates of violent crime [4, 33], so we expected similar associations for LAD-level Agreeableness. Conscientiousness reflects individual differences in organization and self-discipline, and is positively linked to conservative political orientation, academic ability, and income [39, 40]. There is some evidence that aggregate-level Conscientiousness is positively related to conservatism [32], so we expected similar associations in the present investigation, but analyses of nations and states have failed to show consistent findings linking aggregate Conscientiousness to education or income, so we made no predictions about how LAD-level Conscientiousness would relate to the economic indicators. Neuroticism reflects individual differences in anxiety and depression and is associated with indicators of poor health at individual and aggregate levels of analysis [4, 35, 40], so we expected LAD-level Neuroticism to be negatively associated with health indicators. Openness reflects individual differences in curiosity and liberal values, and aggregate-level Openness has been linked to votes for liberal political candidates, human capital, wealth, and social tolerance [4, 32, 41], so we expected LAD-level Openness to be related to indicators of liberalism, economic prosperity, and social diversity.

## Methods

Our analyses were based on data from a large Internet-based survey designed and administered in collaboration with the British Broadcasting Corporation (BBC). Between November 2009 and April 2011, 588,014 individuals competed the “Big Personality Test,” which consisted of eight sections covering demographics, education and work, personal relationships, personality and aspirations, health, and childhood experiences (see S1 Survey). For the present investigation, our analyses focused only on select demographic variables and the personality measures.

### Participants and procedure

The Psychology Research Ethics Committee of the University of Cambridge approved the research and procedure for obtaining consent in October 2007. Volunteers were told that the survey was designed to assess personality and that by clicking on the link to proceed to the survey they were giving their consent to participate. Informed consent was not requested from the next of kin, caretakers, or guardians on behalf of minors or children because only individuals 18 and older were eligible to participate. Initiating the survey was used as a record of participant consent.

The survey was advertised and promoted through various BBC websites, radio programs, and television shows. To complete the survey, respondents clicked on a link on the BBC’s *Lab UK* website. Volunteers were told that the survey was designed to assess personality. Before beginning the survey, respondents were asked to create a BBC ID if they did not already have one. This was used to invite participants to take part in future projects and to prevent individuals from repeat responding—the survey could not be completed more than once with the same ID. After completing the survey, participants received customized feedback about their personalities based on their responses to the survey items.

The central aim of the current investigation was to map the distribution of personality in Great Britain, so of all the participants who completed the survey, we only included those who reported living in England, Wales, Scotland, or Northern Ireland. However, the sample sizes for the districts in Northern Ireland were generally small, so to avoid generating unreliable personality estimates participants from Northern Ireland were excluded from the analyses. Participants who did not complete the personality measure were also excluded. These selection criteria resulted in a total sample of 386,375 respondents (64% female). The mean age of respondents was 35.98 years (*SD* = 13.86 years). Of those who provided information about each of the demographic variables, 13,744 respondents (4%) were Asian; 4,883 (1%) were Black; 8,265 (2%) were of mixed ethnicity; 344,560 (92%) were White; and 3,759 (1%) indicated “Other.” For the education, employment, and income variables, 171,033 (45%) participants reported completing an undergraduate or postgraduate degree, 253,925 (82%) reported being employed full time, part time, or self-employed, and 155,794 (51%) reported earning between £9,999 up to £29,999 per annum and 147,942 (49%) reported earning £30,000 or more per annum. Descriptive statistics for the sample are reported in Table 1.

**Table 1:** Sample characteristics.

	*N*	%
**Gender**		
**Male**	138,820	36%
**Female**	247,551	64%
**Age**		
**19 and younger**	36,700	10%
**20 to 29**	116,262	30%
**30 to 39**	90,059	23%
**40 to 49**	73,393	19%
**50 to 59**	44,218	11%
**60 to 69**	21,407	6%
**70 and older**	4,336	1%
**Ethnicity**		
**Asian**	13,744	4%
**Black**	4,883	1%
**Mixed**	8,265	2%
**White**	344,560	92%
**Other**	3,759	1%
**Education**		
**No GCSE/CSE/O-Levels**	14,362	4%
**GCSE/CSE/O-Levels**	61,412	16%
**Post-16 vocational course**	21,310	6%
**A-Levels**	53,673	14%
**Undergraduate degree**	107,101	28%
**Postgraduate degree**	63,932	17%
**Still in education**	64,582	17%
**Employment status**		
**Full-time employment**	176,429	57%
**Part-time employment**	48,240	16%
**Self employed**	29,256	9%
**Homemaker/full-time parent**	18,278	6%
**Unemployed**	16,039	5%
**Retired**	22,164	7%
**Annual personal income**		
**Up to £9,999**	35,178	12%
**£10,000 to £19,999**	58,310	19%
**£20,000 to £29,999**	62,306	20%
**£30,000 to £39,999**	47,109	15%
**£40,000 to £49,999**	32,131	11%
**£50,000 to £74,999**	39,619	13%
**£75,000 or more**	29,083	10%
**Country of residence**		
**England**	335,114	86%
**Scotland**	33,353	9%
**Wales**	17,908	5%

Participants reported the country and postcode in which they lived at the time in which they completed the survey. Across Great Britain, 335,114 (86%) participants lived in England, 33,353 (9%) lived in Scotland, and 17,908 (5%) lived in Wales. Using the first half of participants’ postcodes, we determined the Local Authority District (LAD) in which participants lived. The LAD sample sizes ranged between 28 participants from The Isles of Scilly and 5,588 participants from Leeds (mean sample size = 1,023; median = 817).

To evaluate the representativeness of the samples from each of the LADs, we compared the demographic characteristics of the LAD samples with LAD data from the 2011 UK Census. Specifically, we correlated the percentage of respondents in each demographic group from the Internet sample with the percentage of the population from that group within each LAD. The correlation between the number of respondents in a LAD and the population of the LAD was .84, indicating that LADs were well represented in the data. The correlation between the median age of participants and LADs was .79, suggesting similar age patterns in the sample and Census estimates. With regard to ethnicity, the correlations for Asian, Black, Mixed, and White ethnicities were .93, .92, .84, and .95, respectively. Overall, these results suggest that the LAD samples were fairly representative of the local populations.

### Measures

#### Personality

The Big Five Inventory was used to assess personality (BFI; 42). The BFI consists of 44 short statements designed to assess the prototypical traits defining each of the Five Factor Model dimensions: Extraversion, Agreeableness, Conscientiousness, Neuroticism, and Openness. Using a 5-point Likert-type rating scale with endpoints at 1 (*Disagree strongly*) and 5 (*Agree strongly*), respondents indicated the extent to which they agreed with each statement. Consistent with previous research [42], a principal components analysis (PCA) with varimax rotation using the current data revealed five components with the items corresponding to each personality dimension loading on the same factors. The factor loadings for the BFI are shown in S1 Table. Analyses of the BFI scales revealed satisfactory internal reliability (*α*s = .86, .77, .83, .83, and .79, for Extraversion, Agreeableness, Conscientiousness, Neuroticism, and Openness, respectively). Descriptive statistics for the measure are presented in Table 2 and scale inter-correlations at both individual and LAD levels are in Table 3.

**Table 2:** Descriptive statistics for personality traits.

	***N***	***M***	***SD***	***α***	***ICC2***	***Moran’s I***
**Extraversion**	386,375	3.24	0.82	.86	.71	.46
**Agreeableness**	386,375	3.74	0.62	.77	.65	.49
**Conscientiousness**	386,375	3.65	0.70	.83	.86	.43
**Neuroticism**	386,375	2.97	0.81	.83	.60	.42
**Openness**	386,375	3.67	0.64	.79	.93	.56

*Note*. *ICC* = Intraclass Correlation.

**Table 3:** Correlations among personality traits at the individual and LAD levels of analysis.

	1	2	3	4	5
**1. Extraversion**		.13	.12	-.34	.21
**2. Agreeableness**	-.20		.25	-.30	.05
**3. Conscientiousness**	-.05	.52		-.22	-.02
**4. Neuroticism**	-.46	-.25	-.44		-.08
**5. Openness**	.43	-.38	-.39	-.16	

*Note*. Correlations above the diagonal are at the individual level and correlations below the diagonal are at the local authority level. *N*s = 386,372 and 380 for individual and LAD levels, respectively.

### Secondary Data

#### Demographic indicators

Population statistics were obtained from the 2011 U.K. Census [43–47]. Specifically, to measure the demographic composition of LADs, we gathered population estimates for gender, ethnicity, country of birth, and age of residents.

#### Political indicators

Votes cast in the 2005 and 2010 General elections were used as political indicators. Electoral data were obtained from the Electoral Commission [48]. In the present study, we calculated the share of votes cast for Conservative, Labour, and Liberal Democrat candidates from the total number of votes cast in each election.

#### Economic indicators

Wealth and human capital data were used as economic indicators. For wealth, we obtained estimates of median annual income for 2011 from the Office of National Statistics (ONS) Annual Survey of Hours and Earnings [43], which is based on a sample of employee jobs taken from HM Revenue & Customs (HMRC) records. Human capital was based on the level of education and occupational status of residents. Specifically, we used the qualifications data from the 2011 Census to measure the proportion of residents with at least a bachelor’s degree (i.e., level 4 qualifications and above). We also gathered occupational data for the occupational characteristics of residents aged 16 to 74 years. Residents were classified as working in managerial and professional occupations, service and administrative occupations, or trade and elementary occupations.

#### Social indicators

To measure the social characteristics of regions, we gathered information about cultural diversity, marital status, and crime from the 2011 Census. For an indicator of cultural diversity, we computed the proportion of foreign-born residents using country of birth data. The proportion of residents 16 and older who were married was used as an indicator of the degree of social stability within the region. The proportion of residents in a registered same-sex civil partnership was used as an indicator of social tolerance. As an indicator of criminal activity, we used reported incidents of violent crimes committed against persons in 2010 and 2011 from the ONS Region and Country Profiles [44].

#### Health indicators

Six indicators were used to assess the health of regions. Life expectancies at birth for 2008 to 2010 were obtained for both men and women from the ONS. The proportion of residents who reported having a long-term health problem or disability that limited their day-to-day activities for at least one year was used as an indicator of long-term health problems [44]. We gathered mortality statistics for stroke, cancer, and heart disease from the National Health Services. For all three mortality indicators, we used the directly age-standardized rates for all ages during the 2010 to 2012 period [49].

## Results

### Psychometric Analyses

To effectively examine the psychological characteristics of regions in Great Britain, it was essential that we first evaluate the psychometric characteristics of the psychological data across levels of analysis. Doing so ensures the appropriateness of aggregating psychological variables to the LAD level and also reduces the possibility of making incorrect inferences from the data. Thus, before undertaking the focal analyses, psychometric analyses were performed. First, to ensure that the scales assessed the same latent constructs across regions, we examined multiple group measurement invariance. Second, to gauge the degree of sampling error in the LAD-level psychological variables, random-intercept multilevel regression models were examined.

Geographic analyses of psychological traits might be biased if the measurement scales functioned differently in different regions. We therefore examined the measurement invariance of personality traits across locations to test whether the same latent traits were similarly assessed in different locations. To reduce the number of models and to ensure sufficient sample sizes, these analyses were fitted at the level of counties (*n* = 128) rather than local authorities. We fitted a sequence of 128 two-group confirmatory factor analyses for each trait. The factor structure of the trait in each county was compared against the factor structure in the second group, which comprised the other 127 counties. We examined both metric invariance (equal factor loadings) and scalar invariance (equal factor loadings and intercepts). Differences in the comparative fit index (CFI) between constrained and unconstrained models were used to evaluate invariance, with model differences of CFI larger than 0.01 considered as significant deviations from invariance [50]. Thus, there were 1,280 tests of invariance (128 counties X 5 traits X 2 invariance definitions). None of the model comparisons revealed CFI differences of 0.01 or greater, suggesting no local deviations from the overall factor structure of personality traits.

The group-mean reliabilities (also known as the intraclass correlation 2, ICC2; see [51]) of the traits aggregated at the level of local authorities are shown in the penultimate data column in Table 2. The average group-mean reliability of the traits was .75, indicating acceptable reliability of the aggregated local-authority means—although there were lower reliability estimates for Neuroticism (.60) and Agreeableness (.65). The overall degree of spatial clustering of the traits was assessed with Moran’s *I* measure of spatial autocorrelation, which estimates how similar neighboring local authorities are to each other compared to non-neighboring local authorities, and is shown in the last data column in Table 2. Using queen’s adjacency spatial matrix (i.e., neighbors were defined as those local authorities whose boundaries, or corner points, touch each other), the average spatial autocorrelation of the traits was .47, indicating moderate spatial clustering. That is, geographically close local authorities had more similar scores of personality traits than their more distant, non-neighboring local authorities.

In summary, our analyses of the psychometric characteristics of the psychological scales indicated that there were no obvious or consistent regional differences in any of the scale properties. Given these results, LAD-level personality scores were derived from the means of the unit-weighted scale scores of respondents who reported living in a local authority at the time in which they participated in the study. All subsequent analyses of the personality traits were based on the aggregate LAD scores. LAD-level Personality Trait T-Scores are presented in S2 Table.

### Mapping Regional Variation in Personality

The second wave of analyses focused on the geographical variation of scores on the psychological indicators. Specifically, we applied the Getis-Ord G* statistic for geographical clustering analysis (also known as “hotspot analysis”) to locate geographical concentration or concentrations of high and low values of the LAD means. The G* statistic identifies areas that have high (or low) values and that also have neighboring areas that have high (or low) values in the psychological indicators [52]. The statistic is interpreted as a *z*-score, with values above 1.96 or below -1.96 indicating statistically significant clustering. This analysis was performed using the spdep package of R 2.15.1 [53], with contiguity spatial weight matrix (0 = does not share boundary, 1 = shares boundary). (The corresponding maps of raw mean personality scores instead of G statistics are presented in S1 Fig.)

**Fig 1:**
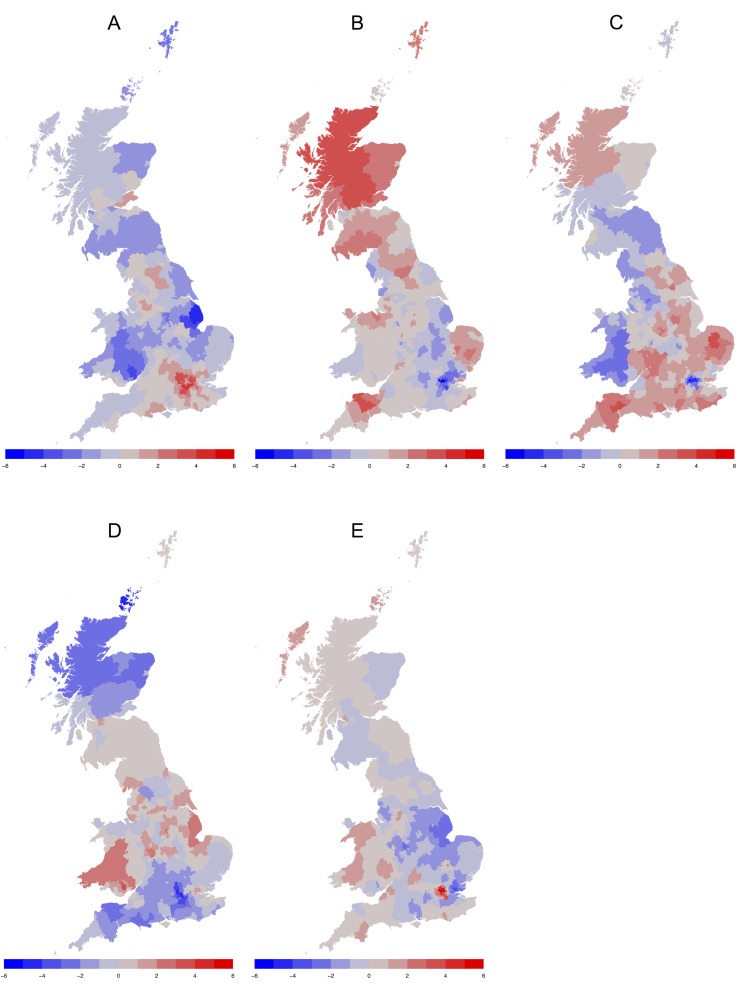
Heat maps of the geographical distribution of personality in Great Britain by LAD. (A) Regional differences in Extraversion. (B) Regional differences in Agreeableness. (C) Regional differences in Conscientiousness. (D) Regional differences in Neuroticism. (E) Regional differences in Openness. For each personality trait, the areas in blue are comparatively low and the areas in red are comparatively high.

The maps displayed in Fig. 1 show geographical concentrations of each of the Big Five traits across Great Britain. What is striking about the maps is that each of the personality traits showed distinct geographical patterns. As can be seen in Fig. 1A, high levels of Extraversion were concentrated in London, various districts in the South and South East of England, Yorkshire, Manchester, and pockets of Scotland, suggesting that large proportions of residents of these areas were social, talkative, and energetic. In contrast, significantly low levels of Extraversion were concentrated in the East Midlands, Wales, Humberside, the North of England, and councils in East Scotland, suggesting that large proportions of residents of these areas were quiet, reserved, and introverted.

The distribution of Agreeableness is presented in in Fig. 1B. The map clearly shows concentrations of high Agreeableness throughout most of Scotland, as well as areas in the North, South West, and East of England, suggesting that disproportionate numbers of residents of these areas were friendly, trusting, and kind. Low levels of Agreeableness were concentrated primarily in London and various districts throughout the East of England, suggesting that comparatively large proportions of residents of these areas were uncooperative, quarrelsome, and irritable.

Fig. 1C shows the geographical distribution of Conscientiousness. The map reveals significantly high levels of Conscientiousness throughout much of Southern England, pockets of the Midlands, and the Scottish Highlands, suggesting that large proportions of residents of these areas were self-disciplined, cautious, and compliant. Significantly low levels of Conscientiousness appeared in London, Wales, and parts of the North of England, suggesting that comparatively large proportions of residents of these areas were disorderly, rebellious, and indifferent.

As can be seen in Fig. 1D, clear clustering of Neuroticism emerged across Great Britain. Significantly high levels of Neuroticism appeared throughout most of Wales and in a number of districts throughout the Midlands, suggesting that large proportions of residents in these areas were comparatively anxious, depressed, and temperamental. Low levels of Neuroticism emerged throughout the South West and much of Southern England, as well as across most of Scotland, suggesting that a disproportionate number of residents of these areas were calm, relaxed, and emotionally stable.

Concentrations of high Openness appeared mainly in metropolitan areas. As can be seen in Fig. 1E, significantly high levels of Openness emerged throughout the London boroughs, Oxford, Cambridge, Brighton, Bristol, Manchester, Glasgow, and parts of Wales, indicating that a disproportion number of residents of these areas were creative, unconventional, and curious. Significantly low levels of Openness emerged throughout most of the East Midlands and East of England, suggesting that large proportions of residents of these areas were conventional, down-to-earth, and traditional.

### Correlations Between LAD-level Personality and PESH Indicators

The maps clearly show regional differences for the personality traits, but how consequential were those differences? Were the regional psychological characteristics linked to important PESH outcomes? To address these questions, the third wave of analyses focused on the associations between the LAD-level personality and PESH indicators. Specifically, we examined the Pearson correlations between the LAD-level personality scores and the PESH indicators. Additionally, because there is robust evidence for gender and age differences in personality [40, 54] and for the impact of income and age on many of the PESH outcomes [55, 56], we also conducted partial correlations controlling for ONS estimates for median income, and median age, and the proportion of female residents derived from the BBC survey data. To avoid focusing on small and potentially unreliable findings, we set our threshold for significance at *r* ≥ .20 and *p* < .001.

As can be seen in Table 4, each of the Big Five personality domains was associated with the various PESH indicators. More specifically, as shown in the first data column, LAD-level Extraversion was associated with high levels of education, income, and high-status occupations, social diversity, and long life expectancies for men and women. Moreover, as shown in the second data column, when gender, age, and income were taken into account, many of the associations with the economic, social, and health indicators remained statistically significant, suggesting that the links between aggregate Extraversion and the indicators were not driven by demographic characteristics.

**Table 4:** Associations between Personality and Demographic, Political, Economic, Social, and Health indicators at the LAD.

	Extraversion		Agreeableness		Conscientiousness		Neuroticism		Openness	
Geographical indicators	***r***	***pr***	***r***	***pr***	***r***	***pr***	***r***	***pr***	***r***	***pr***
***Demographic***										
**Age**	**-.20**		**.47**		**.60**		**-.22**		**-.31**	
**Female**	-.05		**.27**		.19		-.01		-.16	
**Caucasian**	**-.28**	-.14	**.46**	.14	**.50**	.05	-.07	.10	**-.42**	-.16
***Political*** ^**a**^										
**Conservative 2005**	.13	.14	.03	**-.21**	**.51**	**.24**	**-.30**	-.07	-.16	-.08
**Conservative 2010**	.11	.12	.03	**-.20**	**.51**	**.28**	**-.27**	-.04	-.18	-.11
**Labour 2005**	-.16	**-.21**	-.08	.16	**-.45**	-.12	**.42**	**.29**	-.07	**-.23**
**Labour 2010**	-.09	-.16	-.12	.17	**-.54**	**-.23**	**.40**	**.25**	.07	-.11
**Liberal Democrat 2005**	.13	.16	-.04	-.14	.08	-.12	**-.25**	**-.25**	**.28**	**.39**
**Liberal Democrat 2010**	.10	.14	-.05	-.19	.11	-.13	**-.24**	**-.23**	**.24**	**.37**
***Economic***										
**Median annual income in 2011** ^**b**^	**.35**		**-.48**		**-.26**		-.14		**.42**	
**Proportion of residents with higher degree**	**.55**	**.41**	**-.34**	-.14	-.10	.02	**-.41**	**-.41**	**.65**	**.53**
**Managerial and professional occupations**	**.61**	**.48**	**-.36**	-.18	-.01	.12	**-.41**	**-.37**	**.53**	**.40**
**Service and administrative occupations**	**-.33**	**-.22**	**.23**	**.20**	-.08	.06	**.35**	**.24**	**-.51**	**-.49**
**Trade occupations**	**-.62**	**-.51**	**.36**	.13	.05	-.18	**.35**	**.36**	**-.43**	**-.27**
***Social***										
**Foreign-born residents**	**.38**	**.21**	**-.52**	-.19	**-.47**	-.05	-.02	-.18	**.56**	**.29**
**Married residents**	-.18	-.05	**.49**	.15	**.72**	**.29**	**-.29**	-.06	**-.52**	**-.29**
**Same-sex partnerships**	**.35**	.18	**-.56**	**-.31**	**-.43**	-.19	.06	.02	**.62**	**.42**
**Violent crime** ^**c**^	.07	-.10	**-.45**	-.11	**-.53**	-.16	**.21**	.14	**.35**	.09
***Health***										
**Male Life expectancy** ^**d**^	**.26**	**.25**	.00	-.15	**.46**	**.34**	**-.41**	**-.26**	.03	.10
**Female Life expectancy** ^**d**^	**.32**	**.30**	-.06	-.19	**.37**	**.28**	**-.40**	**-.28**	.14	**.20**
**Long-term health problems**	**-.36**	**-.25**	.17	.09	**-.20**	**-.31**	**.42**	**.36**	**-.20**	-.08
**Stroke mortality** ^**e**^	**-.24**	-.15	-.02	-.08	-.15	**-.20**	**.26**	.17	-.08	-.02
**Cancer mortality** ^**e**^	**-.25**	**-.26**	-.06	.10	**-.44**	**-.26**	**.48**	**.37**	-.11	**-.23**
**Heart disease mortality** ^**e**^	**-.24**	**-.24**	-.03	.11	**-.40**	**-.25**	**.43**	**.31**	-.11	-.19

*Note*. E = Extraversion, A = Agreeableness, C = Conscientiousness, N = Neuroticism, O = Openness. *pr* = partial correlations controlling for ONS estimates for median income and BBC estimates for median age and proportion of females per LAD. Coefficients in bold are statistically significant at *p* < .001. *N* = 380.

^a^N = 325.

^b^N = 368.

^c^N = 348.

^d^N = 378.

^e^N = 326.

As can be seen in the third data column, regional differences in Agreeableness were significantly related to the demographic, economic, and social indicators. Specifically, regional Agreeableness was positively associated with median age, proportions of females, Caucasians, low-income residents, service and skilled workers, and married couples, and negatively related to the proportions of university-educated residents, high-status professionals, foreign-born residents, gay couples, as well as rates of violent crime. However, the partial correlations shown in the fourth data column indicate that the magnitude of those associations dropped considerably when gender, age, and income are controlled. Taken together, it appears that the associations between LAD-level Agreeableness and the economic and social indicators were largely driven by the financial and demographic characteristics of residents.

The correlation coefficients in the fifth and sixth data columns show associations between LAD-level Conscientiousness and the PESH indicators. As with Agreeableness, regional Conscientiousness was positively related to age and the proportion of Caucasian residents. Conscientiousness was also associated with several political, social, and health indicators. Specifically, Conscientiousness was positively related to votes for Conservative candidates in the 2005 and 2010 General Elections, the proportion of married residents, and longevity, and negatively correlated with votes for Labour candidates, median income, diversity, violent crime, and deaths from cancer and heart disease. By and large, these associations remained even after controlling for gender, age, and income, suggesting that the associations between LAD-level Conscientiousness and the political and health indicators did not only reflect the demographic characteristics of the residents. In general, this pattern of results suggested that regions with disproportionate numbers of highly conscientious residents were conservative and healthy.

The correlations between LAD-level Neuroticism and the PESH indicators are shown in the seventh and eighth data columns. Regional Neuroticism was significantly associated with most of the political, economic, and health indicators. Specifically, Neuroticism was positively related to votes cast for Labour candidates in the General Elections, the proportion of residents in service and trade professions, violent-crime rates, the proportion of residents reporting long-term health problems, and deaths from stroke, cancer, and heart disease, and it was negatively related to age, votes for Conservative and Liberal Democrat candidates, education level, proportion of high-status professionals, married couples, and life expectancies for men and women. Furthermore, the magnitudes of the associations with most of the political, economic, and health indicators remained significant after controlling for gender, age, and income. Overall, these results indicate that regions with large proportions of people high in Neuroticism had more residents who were politically left-of-center, working class, and physically unhealthy.

The last two data columns show the LAD-level correlations for Openness. Regional Openness was linked to various political, economic, and social indicators. Closer inspection of the results reveals that Openness was positively related to vote shares for Liberal Democrats, residents with university education, income, prevalence of high-status professionals, foreign-born residents, same-sex couples, and rates of violent crime, and negatively associated with age, proportions of Caucasians, service and trade workers, and married couples. Moreover, the majority of these associations remained significant even after controlling for gender, age, and income. Taken together, these results suggested that regions with large numbers of highly open people were cosmopolitan, economically prosperous, and liberal.

## Discussion

### Summary of results

The aim of the current project was to explore the psychological landscape of Great Britain and the associations between regional personality differences and PESH characteristics. Analyses of the psychometric properties of the personality measures revealed no systematic differences between geographic regions. Maps of the personality distributions showed unique clustering for each of the Big Five personality domains with each region uniquely related to the PESH indicators. Taken together, the current results indicate that there are robust regional personality differences in Great Britain and that those differences are meaningful, in so far as they were linked to a diverse spread of PESH outcomes. These results have the potential to inform our understanding of the geographical representation of personality and the mechanisms that contribute to their expression.

### Conceptualizing Aggregate-level Personality Traits

The present findings are generally consistent with results obtained from regional comparisons within the U.S. [3, 4, 32] and with research on the behavioral correlates of personality at the individual level [40, 57]. In the light of these convergent findings, we can begin to develop a solid foundation for conceptualizing region-level personality traits.

Individual-level conceptualizations of Extraversion emphasize assertiveness, energy, enthusiasm, and sociability, and numerous studies have documented links between Extraversion and well being, physical health, leadership, and occupational performance [57]. The results from the current investigation revealed patterns of results that are generally consistent with individual level results. LAD-level Extraversion was most strongly associated with the economic indicators, followed by the health indicators (mean |*pr*| = .32, .24, respectively). Importantly, investigations in the U.S. have also found significant associations between state-level Extraversion and economic and health indicators [4]. Taken together, this pattern of associations suggests that aggregate-level Extraversion reflects the degree to which residents of an area are enterprising and physically healthy.

At the individual level, Agreeableness reflects cooperation, friendliness, and trust, and several studies indicate that such traits are positively associated with prosocial behavior, volunteerism, relationship satisfaction, and nonviolence [40, 57]. Studies in the U.S. have found that state-level Agreeableness is positively associated with social capital and the prevalence of married couples, and negatively associated with social tolerance and crime [3, 4, 32]. Consistent with past work, the most robust pattern of correlations to emerge for LAD-level Agreeableness was with the proportion of same-sex couples. However, the association between LAD-level Agreeableness and crime dropped below our threshold for significance when gender, age, and income were held constant, suggesting that, at least in Great Britain, said association is driven largely by the demographic characteristics of regions. Overall, these findings suggest that aggregate-level Agreeableness reflects the degree to which the residents of a region are communal and conventional.

Research at the individual level indicates that Conscientiousness reflects dutifulness, responsibility, and self-discipline, and that it is positively associated with career success, educational success, longevity, and conservatism [40, 57]. In the current project, LAD-level Conscientiousness was strongly associated with the health, political, and social indicators (mean |*pr*| = .27, .19, .17, respectively). This pattern of results is generally consistent with analyses of state-level Conscientiousness. Based on these findings, it appears that aggregate-level Conscientiousness reflects the degree to which residents of an area are politically and socially conservative, nonviolent, and physically healthy. It is worth noting that national level comparisons of Conscientiousness have not revealed consistent relations with any of the PESH indicators [58].

Individual-level conceptualizations of Neuroticism emphasize anxiety, depression, instability, and stress, and several studies have shown that such traits are negatively associated with relationship satisfaction, psychological well being, career success, effective coping, and longevity [40, 57]. These associations are consistent with findings from analyses of regional differences in the U.S. [4] and also with the present results. Across the PESH indicators, LAD-level Neuroticism was most strongly related to the health indicators (mean |*pr*| = .29), followed by the economic and political indicators (mean |*pr*| = .28, .19, respectively). These findings suggest a conceptualization of aggregate Neuroticism that centers on psychological and physical health. Thus, in regions where there are large proportions of individuals with a disposition toward anxiety and instability, there appear to be large proportions of physically unhealthy and economically disadvantaged residents.

At the individual level, Openness represents creativity, curiosity, imagination, and intellect, and it is positively associated with pursuing a career that involves creativity, living an unconventional lifestyle, earning a college degree, and supporting liberal attitudes [57, 59]. LAD-level Openness was related to support for Liberal Democrats, human capital, proportion of high-status professionals, and cultural and social diversity. This pattern of results is very consistent with findings obtained in U.S. samples and also with individual-level conceptualizations of Openness. Taken together, these results suggest that aggregate Openness represents the degree to which residents are liberal, non-traditional, and educated.

### Implications and Future Directions

A central aim of the current investigation was to replicate and extend previous research on regional personality differences in the U.S. Although it is not possible to make direct comparisons of the geographical distributions of traits in different countries, there are broad similarities in the geographical distributions of some of the Big Five traits across countries. For example, the regions in the U.S. and Great Britain where Agreeableness is highest (i.e., Great Plains and Southern U.S., and Scottish Highlands and the North of England) are generally more rural than urban and have low population densities. In both countries, the regions where Neuroticism is low (the West Coast in the U.S., and the Southwest of England) are generally warmer. And in both the U.S. and Great Britain, the regions high in Openness (Mid-Atlantic and West Coast in the U.S., and London, Brighton, Manchester, and Bristol in England) are more urban and densely populated.

The similar geographical patterns raise interesting questions about the nature of regional personality differences. Specifically, do regional personality differences emerge as a result of individuals with certain traits selectively migrating to places with particular features? Or do differences emerge as a result of some form of social or ecological influence? Although there is currently no longitudinal personality data available that would allow for empirically investigating these questions, there are good reasons to believe that different mechanisms might contribute to the geographical distribution of traits.

Recent research on selective migration indicates that high Openness is associated with moving from one’s home state to a different state and that high Agreeableness is associated with staying within one’s hometown [9, 11, 23]. There is also evidence that Extraversion is associated with a heightened migration propensity, and that residential mobility has adverse health consequences for people low on that trait [9, 12, 13]. These findings provide clues for interpreting the nature of regional personality differences in Openness, Agreeableness, and Extraversion. Perhaps regional differences in Openness are a result of people moving to places where they can satisfy their needs for stimulation and enrichment, which is why Openness is higher in regions rich in human capital. It is conceivable that agreeable individuals’ desire to settle near family and friends is the reason why rates of social capital are high and crime is generally low in high Agreeable regions. Longitudinal studies that assess personality, values, and migration decisions would greatly inform our understanding of the impact of selective migration on regional personality differences. However, it is worth pointing out that the impact of selective migration on regional differences is limited to countries with high rates of internal migration.

Research on social influence suggests that mood and emotion are susceptible to social influence. For example, research indicates that the positive affect of one’s friends influences individuals’ levels of happiness [60] and that the negative affect of intimate partners can increase individuals’ levels of depression [61]. These findings make it reasonable to hypothesize that social influence might be the mechanism responsible for regional differences in Neuroticism. Consider, for instance, living in a place where there is a large proportion of anxious and irritable residents. The current findings raise the question of whether living in such a place would increase the level of negative affect of the people who live in that area and lead to a high level of Neuroticism for the area. Add to that the poor physical health that is common in high Neuroticism regions, and residents would have good reasons to be anxious and depressed. Research that evaluates personality change within a regional context—that is, whether traits change in response to one’s local social environment—has the potential to inform our understanding of the possible role social influence has on regional personality differences, as well as on personality development.

It is also conceivable that such factors as climate, terrain, and ethnic diversity influence the activities people pursue, their lifestyles, and how they relate to each other. In this way, features of the environment can affect the prevalence of certain traits. Work by Schaller and colleagues [15–17] suggests that national levels of Openness and Extraversion are low in regions where rates of infectious disease are historically high because limited social contact and exposure to novel stimuli reduce the spread of illnesses. Those findings raise the possibility that ecological influences might be responsible for regional differences in Openness and Extraversion. It is conceivable that living in a stimulating environment where individuals are regularly challenged with new experiences could encourage open-mindedness, curiosity, assertiveness, and self-confidence. Research that examines the extent to which features of the environment influence personality could determine whether ecological influences contribute to regional psychological differences.

Taken together, the current evidence makes it reasonable to hypothesize that certain mechanisms might affect some traits more than others. It appears that regional differences in Openness could be driven by selective migration, social influence, and ecological influence. Social influence and possibly ecological influence appear to be the forces contributing to differences in Neuroticism. Regional differences in Agreeableness would seem to be driven mainly by selective migration. And selective migration and ecological influence may be the main mechanisms contributing to regional differences in Extraversion. It is interesting to note that none of the current research on the mechanisms underlying regional differences offers clues about the geographic distribution of Conscientiousness. Future research examining the differential effects of the mechanisms on geographic variation in various personality traits could greatly inform our understanding of their impact.

## Conclusion

The current investigation was designed to replicate and extend previous research on regional personality differences by examining the distribution and macro-level correlates of personality in Great Britain. The present results converged with findings from previous studies, thereby providing a solid foundation for developing and testing hypotheses about the dynamic relationships between personality and the places in which people live. The work also raises important questions about the nature of personality, its role in society, and its impact on broad social processes. Further exploration of regional differences in other psychological constructs and processes will help broaden our understanding of the ways in which psychological, political, economic, social, and health factors shape human social behavior.

## Supporting Information

S1 Fig Maps of Average Scores for each Big Five Personality Domain by LAD. For each personality trait, the areas in blue are comparatively low and the areas in red are comparatively high.
(TIFF)

S1 Table Factor Structure of Big Five Inventory. (DOCX)

S2 Table Local Authority District level Personality Trait T-Scores. (DOCX)

S1 Survey The Big Persoanlity Test (DOCX)
