# Supplementary figures and images for: Regional Personality Differences in Great Britain
Source: PLoS One. 2015 Mar 24;10(3):e0122245. doi: 10.1371/journal.pone.0122245 (PMC4372610; doi:10.1371/journal.pone.0122245)

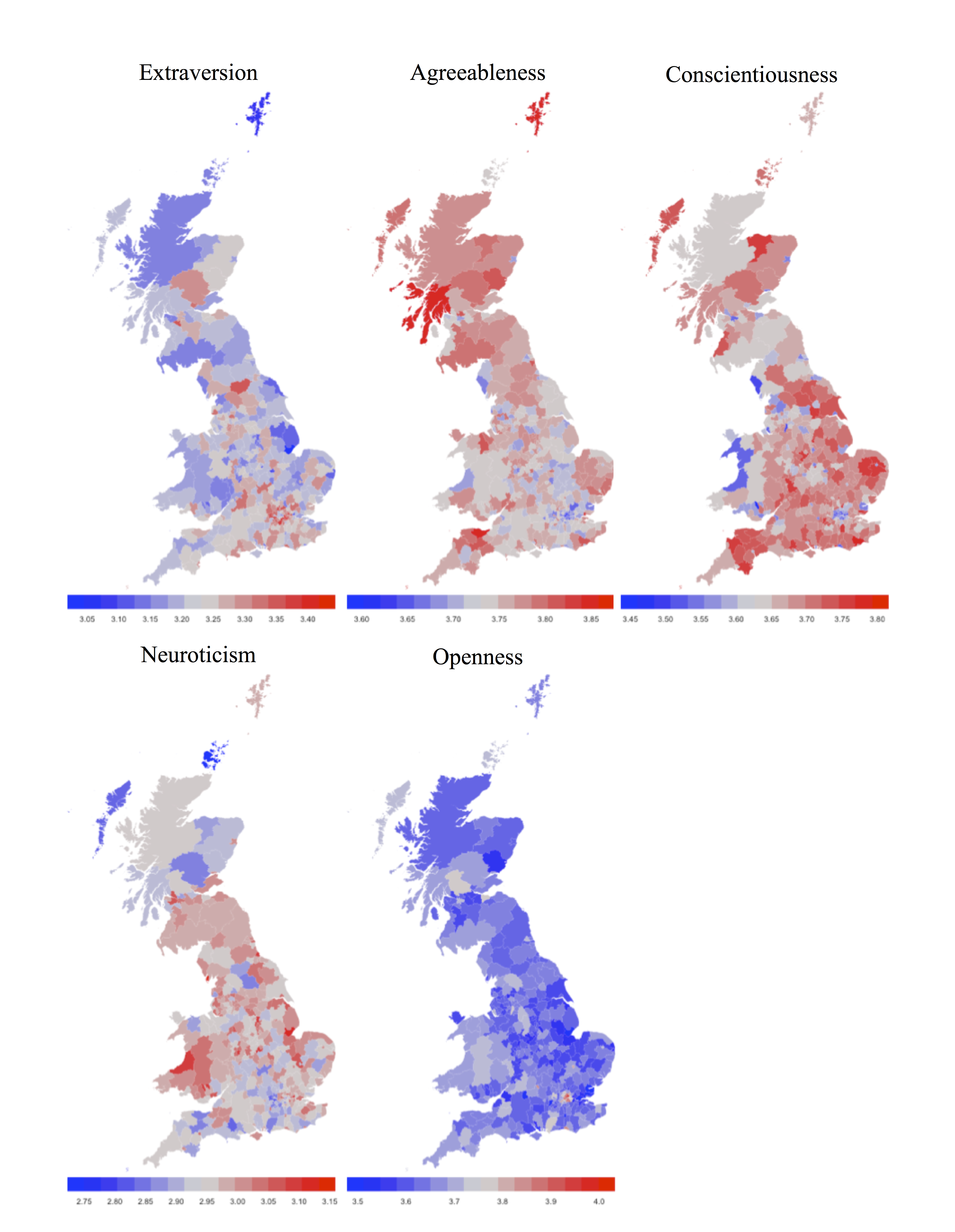

Supplement: S1 Fig — For each personality trait, the areas in blue are comparatively low and the areas in red are comparatively high. (TIFF) [file pone.0122245.s001.tiff]
