# Supplementary material for: Regional Personality Differences in Great Britain
Source: PLoS One. 2015 Mar 24;10(3):e0122245. doi: 10.1371/journal.pone.0122245 (PMC4372610; doi:10.1371/journal.pone.0122245)
Supplement: S1 Table — (DOCX) [file pone.0122245.s002.docx]

**Table S1. Factor Structure of Big Five Inventory**

|  | **Varimax-Rotated Principal Component** | | | | |
| --- | --- | --- | --- | --- | --- |
|  | **1** | **2** | **3** | **4** | **5** |
| **BFI_21 Tends to be quiet (r)** | **.83** | -.03 | -.05 | -.08 | -.02 |
| **BFI_1 Is talkative** | **.78** | -.03 | .08 | .08 | .08 |
| **BFI_6 Is reserved (r)** | **.77** | -.05 | -.04 | -.11 | .02 |
| **BFI_36 Is outgoing, sociable** | **.74** | -.02 | .10 | -.10 | .19 |
| **BFI_31 Is sometimes shy, inhibited (r)** | **.70** | .05 | -.08 | -.24 | -.03 |
| **BFI_26 Has an assertive personality** | **.57** | .20 | .23 | -.19 | -.23 |
| **BFI_16 Generates a lot of enthusiasm** | **.57** | .15 | .37 | -.12 | .17 |
| **BFI_11 Is full of energy** | **.46** | .20 | .23 | -.27 | .12 |
| **BFI_3 Does a thorough job** | .02 | **.73** | .09 | .04 | .06 |
| **BFI_33 Does things efficiently** | .04 | **.71** | .07 | -.06 | .09 |
| **BFI_28 Perseveres until the task is finished** | .01 | **.68** | .14 | -.03 | .07 |
| **BFI_18 Tends to be disorganized (r)** | .00 | **.67** | -.18 | -.04 | -.01 |
| **BFI_38 Makes plans and follows through with them** | .14 | **.64** | .06 | -.06 | .05 |
| **BFI_13 Is a reliable worker** | .03 | **.64** | .00 | .01 | .20 |
| **BFI_23 Tends to be lazy (r)** | .15 | **.62** | -.06 | -.10 | .13 |
| **BFI_43 Is easily distracted (r)** | -.09 | **.59** | -.12 | -.19 | .04 |
| **BFI_8 Can be somewhat careless (r)** | -.11 | **.58** | -.11 | -.09 | .10 |
| **BFI_25 Is inventive** | .10 | .04 | **.71** | -.21 | -.07 |
| **BFI_5 Is original, comes up with new ideas** | .19 | .06 | **.68** | -.17 | -.07 |
| **BFI_20 Has an active imagination** | .13 | -.09 | **.67** | .11 | .02 |
| **BFI_40 Likes to reflect, play with ideas** | -.05 | .03 | **.66** | -.01 | .05 |
| **BFI_15 Is ingenious, a deep thinker** | -.10 | .07 | **.63** | .08 | -.09 |
| **BFI_30 Values artistic, aesthetic experiences** | .01 | -.02 | **.62** | .10 | .15 |
| **BFI_10 Is curious about many different things** | .11 | .00 | **.60** | -.11 | .02 |
| **BFI_44 Is sophisticated in art, music, or literature** | .00 | -.09 | **.58** | .07 | .07 |
| **BFI_41 Has few artistic interests (r)** | -.02 | -.02 | **.41** | .02 | .08 |
| **BFI_35 Prefers work that is routine (r)** | .13 | -.10 | **.29** | -.23 | -.07 |
| **BFI_19 Worries a lot** | -.11 | .00 | .04 | **.76** | .03 |
| **BFI_9 Is relaxed, handles stress well (r)** | -.04 | -.04 | -.11 | **.75** | -.09 |
| **BFI_14 Can be tense** | -.08 | .05 | .08 | **.69** | -.18 |
| **BFI_24 Is emotionally stable, not easily upset (r)** | -.04 | -.12 | -.04 | **.68** | -.09 |
| **BFI_39 Gets nervous easily** | -.31 | -.09 | -.03 | **.62** | .08 |
| **BFI_34 Remains calm in tense situations (r)** | -.01 | -.20 | -.19 | **.60** | -.10 |
| **BFI_4 Is depressed, blue** | -.25 | -.15 | .09 | **.58** | -.18 |
| **BFI_29 Can be moody** | -.07 | -.11 | .07 | **.51** | -.37 |
| **BFI_32 Is considerate and kind to almost everyone** | .02 | .16 | .12 | .07 | **.70** |
| **BFI_37 Is sometimes rude to others (r)** | -.13 | .15 | -.06 | -.11 | **.63** |
| **BFI_17 Has a forgiving nature** | .02 | -.07 | .12 | -.11 | **.60** |
| **BFI_27 Can be cold and aloof (r)** | .32 | .05 | -.11 | -.06 | **.56** |
| **BFI_12 Starts quarrels with others (r)** | -.21 | .13 | -.03 | -.21 | **.54** |
| **BFI_42 Likes to cooperate with others** | .19 | .16 | .04 | .02 | **.54** |
| **BFI_2 Tends to find fault with others (r)** | -.08 | -.04 | -.01 | -.27 | **.54** |
| **BFI_7 Is helpful and unselfish with others** | .05 | .20 | .14 | .02 | **.54** |
| **BFI_22 Is generally trusting** | .11 | .04 | .01 | -.05 | **.51** |

Note. (r) = reverse keyed item.
