# Supplementary material for: Regional Personality Differences in Great Britain
Source: PLoS One. 2015 Mar 24;10(3):e0122245. doi: 10.1371/journal.pone.0122245 (PMC4372610; doi:10.1371/journal.pone.0122245)
Supplement: S2 Table — (DOCX) [file pone.0122245.s003.docx]

**Table S2. Local Authority District level Personality Trait T-Scores**

| **LAD ID** | **LAD Name** | **E** | **A** | **C** | **N** | **O** |
| --- | --- | --- | --- | --- | --- | --- |
| E06000001 | Hartlepool UA | 48.1 | 44.9 | 45.4 | 72.5 | 48.6 |
| E06000002 | Middlesbrough UA | 48.9 | 39.9 | 33.0 | 54.9 | 47.8 |
| E06000003 | Redcar and Cleveland UA | 41.8 | 42.8 | 38.6 | 52.0 | 44.3 |
| E06000004 | Stockton-on-Tees UA | 57.4 | 58.6 | 50.6 | 53.3 | 44.6 |
| E06000005 | Darlington UA | 45.6 | 58.9 | 51.4 | 44.1 | 48.6 |
| E06000006 | Halton UA | 48.2 | 63.1 | 48.7 | 62.0 | 48.6 |
| E06000007 | Warrington UA | 51.5 | 53.8 | 55.8 | 53.3 | 42.3 |
| E06000008 | Blackburn with Darwen UA | 53.6 | 67.1 | 42.3 | 57.9 | 47.0 |
| E06000009 | Blackpool UA | 50.8 | 33.3 | 35.3 | 59.3 | 41.4 |
| E06000010 | Kingston upon Hull, City of UA | 38.2 | 40.7 | 32.3 | 60.9 | 47.6 |
| E06000011 | East Riding of Yorkshire UA | 47.8 | 49.3 | 63.9 | 47.0 | 43.6 |
| E06000012 | North East Lincolnshire UA | 46.9 | 37.7 | 44.0 | 70.1 | 36.6 |
| E06000013 | North Lincolnshire UA | 47.7 | 56.3 | 54.3 | 61.7 | 39.4 |
| E06000014 | York UA | 53.3 | 48.6 | 39.7 | 50.7 | 57.0 |
| E06000015 | Derby UA | 48.7 | 41.1 | 52.0 | 57.8 | 47.8 |
| E06000016 | Leicester UA | 46.7 | 50.2 | 34.2 | 57.8 | 54.3 |
| E06000017 | Rutland UA | 63.4 | 39.6 | 57.2 | 40.9 | 58.7 |
| E06000018 | Nottingham UA | 55.2 | 43.0 | 32.2 | 54.6 | 54.4 |
| E06000019 | Herefordshire, County of UA | 35.7 | 48.7 | 54.3 | 51.2 | 57.3 |
| E06000020 | Telford and Wrekin UA | 34.9 | 56.8 | 47.6 | 55.2 | 41.2 |
| E06000021 | Stoke-on-Trent UA | 38.2 | 38.8 | 40.0 | 68.9 | 47.2 |
| E06000022 | Bath and North East Somerset UA | 49.6 | 46.0 | 40.2 | 47.5 | 59.6 |
| E06000023 | Bristol, City of UA | 56.3 | 40.0 | 31.7 | 52.4 | 64.4 |
| E06000024 | North Somerset UA | 51.4 | 55.5 | 59.6 | 38.0 | 40.7 |
| E06000025 | South Gloucestershire UA | 46.5 | 47.9 | 55.5 | 46.0 | 37.9 |
| E06000026 | Plymouth UA | 52.9 | 53.1 | 45.2 | 55.1 | 47.4 |
| E06000027 | Torbay UA | 61.0 | 53.3 | 61.7 | 37.2 | 57.5 |
| E06000028 | Bournemouth UA | 60.3 | 50.5 | 41.1 | 44.2 | 49.4 |
| E06000029 | Poole UA | 50.7 | 51.6 | 57.4 | 46.9 | 47.5 |
| E06000030 | Swindon UA | 45.2 | 43.4 | 50.4 | 53.7 | 33.0 |
| E06000031 | Peterborough UA | 52.9 | 39.4 | 44.7 | 48.7 | 48.9 |
| E06000032 | Luton UA | 49.8 | 51.0 | 46.1 | 38.6 | 42.9 |
| E06000033 | Southend-on-Sea UA | 53.9 | 60.1 | 53.2 | 48.2 | 48.0 |
| E06000034 | Thurrock UA | 40.5 | 38.5 | 53.4 | 62.6 | 32.2 |
| E06000035 | Medway UA | 44.8 | 57.9 | 50.7 | 48.5 | 40.3 |
| E06000036 | Bracknell Forest UA | 43.3 | 44.5 | 66.1 | 42.6 | 32.0 |
| E06000037 | West Berkshire UA | 52.6 | 50.2 | 54.5 | 46.4 | 46.8 |
| E06000038 | Reading UA | 48.1 | 34.5 | 32.2 | 52.2 | 56.3 |
| E06000039 | Slough UA | 46.8 | 58.8 | 47.3 | 40.4 | 49.7 |
| E06000040 | Windsor and Maidenhead UA | 72.2 | 38.1 | 56.8 | 25.3 | 55.8 |
| E06000041 | Wokingham UA | 60.7 | 48.2 | 59.1 | 41.5 | 43.7 |
| E06000042 | Milton Keynes UA | 49.8 | 48.4 | 55.3 | 50.9 | 50.9 |
| E06000043 | Brighton and Hove UA | 68.0 | 39.4 | 27.9 | 58.5 | 75.1 |
| E06000044 | Portsmouth UA | 52.9 | 39.9 | 37.2 | 51.1 | 56.1 |
| E06000045 | Southampton UA | 50.2 | 42.8 | 41.3 | 55.0 | 52.0 |
| E06000046 | Isle of Wight UA | 45.0 | 51.1 | 56.0 | 55.6 | 51.9 |
| E06000047 | County Durham UA | 46.0 | 57.3 | 46.1 | 55.4 | 51.8 |
| E06000048 | Northumberland UA | 43.4 | 52.1 | 51.0 | 52.0 | 47.3 |
| E06000049 | Cheshire East UA | 55.6 | 52.8 | 55.8 | 43.1 | 48.6 |
| E06000050 | Cheshire West and Chester UA | 46.6 | 55.1 | 49.6 | 45.5 | 47.5 |
| E06000051 | Shropshire UA | 51.7 | 50.2 | 55.5 | 49.9 | 55.3 |
| E06000052 | Cornwall UA | 48.9 | 54.5 | 49.7 | 49.2 | 57.6 |
| E06000053 | Isles of Scilly UA | 59.1 | 87.6 | 77.5 | 16.6 | 64.2 |
| E06000054 | Wiltshire UA | 50.8 | 50.5 | 55.1 | 44.5 | 46.7 |
| E06000055 | Bedford UA | 48.7 | 42.8 | 54.5 | 41.4 | 48.4 |
| E06000056 | Central Bedfordshire UA | 41.2 | 43.6 | 53.0 | 56.5 | 42.3 |
| E07000004 | Aylesbury Vale | 56.7 | 55.8 | 60.5 | 43.8 | 49.4 |
| E07000005 | Chiltern | 62.5 | 50.0 | 50.5 | 43.4 | 51.7 |
| E07000006 | South Bucks | 69.1 | 32.4 | 54.9 | 30.0 | 68.5 |
| E07000007 | Wycombe | 66.9 | 43.4 | 51.5 | 37.3 | 44.0 |
| E07000008 | Cambridge | 46.1 | 46.5 | 42.3 | 58.0 | 74.4 |
| E07000009 | East Cambridgeshire | 37.6 | 59.2 | 55.8 | 68.9 | 43.3 |
| E07000010 | Fenland | 40.4 | 45.6 | 55.0 | 61.6 | 41.5 |
| E07000011 | Huntingdonshire | 49.0 | 45.2 | 61.4 | 40.4 | 40.2 |
| E07000012 | South Cambridgeshire | 42.1 | 44.6 | 56.5 | 47.5 | 58.2 |
| E07000026 | Allerdale | 55.0 | 44.8 | 46.3 | 48.3 | 51.1 |
| E07000027 | Barrow-in-Furness | 42.1 | 43.2 | 37.9 | 83.3 | 42.0 |
| E07000028 | Carlisle | 35.1 | 56.5 | 41.6 | 59.9 | 48.9 |
| E07000029 | Copeland | 33.9 | 30.1 | 26.3 | 60.1 | 55.5 |
| E07000030 | Eden | 47.3 | 53.3 | 61.7 | 45.0 | 46.3 |
| E07000031 | South Lakeland | 54.5 | 51.8 | 46.7 | 53.3 | 50.5 |
| E07000032 | Amber Valley | 41.0 | 49.4 | 60.2 | 48.4 | 48.5 |
| E07000033 | Bolsover | 29.8 | 58.7 | 49.4 | 73.0 | 43.0 |
| E07000034 | Chesterfield | 34.4 | 58.8 | 48.9 | 53.3 | 39.9 |
| E07000035 | Derbyshire Dales | 46.1 | 58.6 | 55.0 | 45.8 | 60.7 |
| E07000036 | Erewash | 51.7 | 60.9 | 57.1 | 48.5 | 41.5 |
| E07000037 | High Peak | 59.2 | 59.8 | 49.9 | 39.7 | 60.4 |
| E07000038 | North East Derbyshire | 45.7 | 58.3 | 49.7 | 46.0 | 41.8 |
| E07000039 | South Derbyshire | 50.8 | 56.2 | 69.4 | 47.6 | 38.1 |
| E07000040 | East Devon | 48.8 | 63.3 | 63.0 | 39.6 | 54.5 |
| E07000041 | Exeter | 53.8 | 46.7 | 41.3 | 61.5 | 53.9 |
| E07000042 | Mid Devon | 40.3 | 67.5 | 64.1 | 30.4 | 50.6 |
| E07000043 | North Devon | 49.9 | 61.9 | 63.3 | 45.7 | 53.0 |
| E07000044 | South Hams | 52.3 | 61.4 | 67.1 | 37.8 | 57.7 |
| E07000045 | Teignbridge | 52.3 | 52.4 | 59.2 | 45.7 | 51.4 |
| E07000046 | Torridge | 41.4 | 66.4 | 68.0 | 41.2 | 45.8 |
| E07000047 | West Devon | 50.1 | 41.7 | 60.9 | 49.4 | 60.0 |
| E07000048 | Christchurch | 61.4 | 56.8 | 62.2 | 31.5 | 49.3 |
| E07000049 | East Dorset | 62.0 | 58.9 | 68.4 | 37.3 | 49.7 |
| E07000050 | North Dorset | 53.2 | 49.0 | 60.0 | 25.9 | 51.5 |
| E07000051 | Purbeck | 60.3 | 48.6 | 52.1 | 43.1 | 46.7 |
| E07000052 | West Dorset | 49.4 | 50.2 | 52.2 | 43.6 | 56.0 |
| E07000053 | Weymouth and Portland | 60.5 | 60.3 | 53.9 | 32.7 | 50.3 |
| E07000061 | Eastbourne | 53.8 | 60.4 | 53.1 | 51.3 | 50.0 |
| E07000062 | Hastings | 35.0 | 42.6 | 52.7 | 65.8 | 54.5 |
| E07000063 | Lewes | 46.9 | 45.2 | 60.7 | 47.4 | 54.1 |
| E07000064 | Rother | 49.8 | 70.9 | 70.5 | 37.7 | 57.7 |
| E07000065 | Wealden | 61.8 | 57.0 | 58.8 | 39.3 | 48.3 |
| E07000066 | Basildon | 47.4 | 44.2 | 49.8 | 51.1 | 33.9 |
| E07000067 | Braintree | 45.5 | 40.5 | 53.2 | 52.1 | 50.3 |
| E07000068 | Brentwood | 63.8 | 43.8 | 55.1 | 51.9 | 47.4 |
| E07000069 | Castle Point | 51.6 | 77.2 | 54.3 | 44.3 | 33.1 |
| E07000070 | Chelmsford | 42.8 | 44.6 | 56.7 | 47.0 | 41.6 |
| E07000071 | Colchester | 50.3 | 56.3 | 46.0 | 45.6 | 45.8 |
| E07000072 | Epping Forest | 69.0 | 26.1 | 42.8 | 52.9 | 45.3 |
| E07000073 | Harlow | 50.7 | 44.1 | 45.0 | 44.6 | 43.2 |
| E07000074 | Maldon | 57.5 | 32.4 | 34.7 | 48.7 | 28.1 |
| E07000075 | Rochford | 53.9 | 57.0 | 66.3 | 42.9 | 29.9 |
| E07000076 | Tendring | 43.9 | 54.2 | 58.0 | 53.5 | 48.6 |
| E07000077 | Uttlesford | 51.6 | 53.5 | 60.5 | 44.0 | 50.9 |
| E07000078 | Cheltenham | 58.5 | 51.5 | 52.5 | 42.3 | 56.1 |
| E07000079 | Cotswold | 65.4 | 52.7 | 58.5 | 38.9 | 60.7 |
| E07000080 | Forest of Dean | 33.5 | 65.3 | 63.7 | 49.4 | 53.0 |
| E07000081 | Gloucester | 49.7 | 54.7 | 51.6 | 47.0 | 40.1 |
| E07000082 | Stroud | 46.1 | 66.1 | 54.3 | 46.5 | 55.1 |
| E07000083 | Tewkesbury | 40.4 | 47.7 | 59.2 | 52.6 | 41.8 |
| E07000084 | Basingstoke and Deane | 47.4 | 47.3 | 54.1 | 52.8 | 43.1 |
| E07000085 | East Hampshire | 52.4 | 55.0 | 61.6 | 46.4 | 45.4 |
| E07000086 | Eastleigh | 40.4 | 63.4 | 62.9 | 41.1 | 36.4 |
| E07000087 | Fareham | 45.6 | 52.5 | 63.2 | 41.3 | 31.7 |
| E07000088 | Gosport | 44.7 | 60.6 | 61.1 | 42.5 | 36.3 |
| E07000089 | Hart | 48.7 | 57.8 | 54.2 | 39.7 | 47.5 |
| E07000090 | Havant | 38.1 | 47.0 | 57.2 | 49.9 | 44.7 |
| E07000091 | New Forest | 56.5 | 50.2 | 57.5 | 49.2 | 42.9 |
| E07000092 | Rushmoor | 59.3 | 41.8 | 52.9 | 61.4 | 44.8 |
| E07000093 | Test Valley | 48.1 | 54.7 | 61.4 | 49.1 | 47.4 |
| E07000094 | Winchester | 62.6 | 53.6 | 54.6 | 41.2 | 54.6 |
| E07000095 | Broxbourne | 47.6 | 54.6 | 66.8 | 48.3 | 41.4 |
| E07000096 | Dacorum | 53.3 | 56.0 | 59.5 | 40.2 | 50.1 |
| E07000097 | East Hertfordshire | 61.2 | 44.4 | 52.7 | 50.3 | 45.8 |
| E07000098 | Hertsmere | 73.0 | 55.5 | 60.0 | 44.4 | 48.0 |
| E07000099 | North Hertfordshire | 46.8 | 49.9 | 54.4 | 44.4 | 55.9 |
| E07000100 | St Albans | 58.2 | 52.3 | 55.4 | 37.7 | 51.8 |
| E07000101 | Stevenage | 40.6 | 59.7 | 63.6 | 45.9 | 41.9 |
| E07000102 | Three Rivers | 74.7 | 56.3 | 49.7 | 43.3 | 51.0 |
| E07000103 | Watford | 59.9 | 40.6 | 46.8 | 47.3 | 52.1 |
| E07000104 | Welwyn Hatfield | 37.9 | 46.2 | 43.8 | 49.2 | 46.8 |
| E07000105 | Ashford | 60.0 | 53.5 | 58.0 | 49.3 | 51.4 |
| E07000106 | Canterbury | 42.5 | 45.9 | 51.9 | 46.9 | 62.9 |
| E07000107 | Dartford | 49.4 | 51.6 | 48.5 | 48.6 | 47.2 |
| E07000108 | Dover | 53.3 | 58.4 | 60.0 | 50.8 | 51.8 |
| E07000109 | Gravesham | 57.8 | 47.1 | 53.2 | 46.0 | 34.4 |
| E07000110 | Maidstone | 51.3 | 49.4 | 44.7 | 62.3 | 48.6 |
| E07000111 | Sevenoaks | 52.8 | 48.7 | 51.7 | 44.1 | 47.7 |
| E07000112 | Shepway | 57.1 | 58.1 | 54.3 | 42.9 | 47.4 |
| E07000113 | Swale | 38.7 | 49.1 | 51.0 | 57.6 | 46.1 |
| E07000114 | Thanet | 45.1 | 55.1 | 46.8 | 58.3 | 48.2 |
| E07000115 | Tonbridge and Malling | 58.0 | 50.7 | 58.1 | 47.2 | 44.5 |
| E07000116 | Tunbridge Wells | 56.8 | 38.7 | 54.7 | 53.1 | 56.0 |
| E07000117 | Burnley | 55.1 | 54.6 | 42.4 | 64.1 | 40.7 |
| E07000118 | Chorley | 56.3 | 40.2 | 55.8 | 50.9 | 45.0 |
| E07000119 | Fylde | 69.2 | 56.8 | 53.9 | 35.9 | 49.2 |
| E07000120 | Hyndburn | 46.3 | 45.5 | 56.0 | 56.9 | 37.7 |
| E07000121 | Lancaster | 37.8 | 51.9 | 38.3 | 57.5 | 57.5 |
| E07000122 | Pendle | 47.3 | 51.9 | 50.5 | 37.3 | 41.7 |
| E07000123 | Preston | 41.8 | 35.7 | 36.5 | 62.8 | 47.4 |
| E07000124 | Ribble Valley | 41.3 | 40.9 | 33.1 | 47.0 | 55.1 |
| E07000125 | Rossendale | 67.4 | 39.7 | 41.0 | 40.9 | 58.4 |
| E07000126 | South Ribble | 49.3 | 62.9 | 46.2 | 60.6 | 43.5 |
| E07000127 | West Lancashire | 48.2 | 57.7 | 46.7 | 61.0 | 43.9 |
| E07000128 | Wyre | 49.5 | 42.5 | 60.3 | 55.2 | 39.8 |
| E07000129 | Blaby | 50.6 | 46.6 | 47.9 | 72.0 | 39.0 |
| E07000130 | Charnwood | 55.0 | 54.1 | 53.1 | 49.1 | 48.2 |
| E07000131 | Harborough | 47.9 | 41.4 | 44.9 | 40.4 | 50.1 |
| E07000132 | Hinckley and Bosworth | 45.0 | 47.1 | 59.7 | 48.5 | 44.7 |
| E07000133 | Melton | 46.0 | 51.5 | 59.6 | 59.6 | 46.4 |
| E07000134 | North West Leicestershire | 43.5 | 46.2 | 55.1 | 45.7 | 41.8 |
| E07000135 | Oadby and Wigston | 30.1 | 57.1 | 64.9 | 68.4 | 50.5 |
| E07000136 | Boston | 11.3 | 44.6 | 39.6 | 79.2 | 33.0 |
| E07000137 | East Lindsey | 30.0 | 51.5 | 57.3 | 58.1 | 45.7 |
| E07000138 | Lincoln | 45.0 | 55.9 | 53.5 | 55.3 | 47.1 |
| E07000139 | North Kesteven | 40.1 | 50.8 | 64.1 | 50.6 | 37.7 |
| E07000140 | South Holland | 44.0 | 45.8 | 57.6 | 64.6 | 37.6 |
| E07000141 | South Kesteven | 46.0 | 50.6 | 56.0 | 43.0 | 42.5 |
| E07000142 | West Lindsey | 28.3 | 41.7 | 53.7 | 44.1 | 49.1 |
| E07000143 | Breckland | 54.8 | 59.9 | 69.3 | 41.2 | 47.2 |
| E07000144 | Broadland | 42.9 | 54.9 | 65.9 | 44.6 | 40.7 |
| E07000145 | Great Yarmouth | 52.2 | 56.4 | 48.8 | 47.3 | 34.8 |
| E07000146 | King’s Lynn and West Norfolk | 41.0 | 59.8 | 53.5 | 59.9 | 43.4 |
| E07000147 | North Norfolk | 44.9 | 47.1 | 53.5 | 57.2 | 54.7 |
| E07000148 | Norwich | 51.0 | 42.9 | 35.7 | 58.6 | 61.9 |
| E07000149 | South Norfolk | 49.3 | 53.4 | 67.0 | 46.7 | 53.7 |
| E07000150 | Corby | 26.5 | 63.2 | 34.7 | 48.6 | 39.0 |
| E07000151 | Daventry | 43.9 | 53.9 | 45.4 | 55.1 | 42.5 |
| E07000152 | East Northamptonshire | 54.4 | 41.1 | 59.0 | 49.1 | 40.8 |
| E07000153 | Kettering | 44.1 | 52.8 | 48.2 | 62.0 | 49.8 |
| E07000154 | Northampton | 45.4 | 48.5 | 49.6 | 50.1 | 42.1 |
| E07000155 | South Northamptonshire | 66.3 | 51.8 | 55.5 | 32.6 | 51.0 |
| E07000156 | Wellingborough | 35.0 | 35.1 | 39.4 | 58.5 | 36.4 |
| E07000163 | Craven | 65.0 | 58.8 | 57.9 | 47.8 | 59.4 |
| E07000164 | Hambleton | 45.3 | 54.7 | 61.7 | 64.6 | 49.0 |
| E07000165 | Harrogate | 56.8 | 61.2 | 61.8 | 32.4 | 50.7 |
| E07000166 | Richmondshire | 70.4 | 62.3 | 57.1 | 37.5 | 43.4 |
| E07000167 | Ryedale | 42.1 | 46.3 | 59.5 | 56.6 | 52.6 |
| E07000168 | Scarborough | 30.0 | 46.5 | 49.6 | 47.7 | 53.0 |
| E07000169 | Selby | 38.8 | 60.3 | 68.2 | 55.0 | 46.7 |
| E07000170 | Ashfield | 36.6 | 51.9 | 49.8 | 65.0 | 45.3 |
| E07000171 | Bassetlaw | 56.2 | 52.8 | 61.2 | 43.2 | 51.9 |
| E07000172 | Broxtowe | 44.6 | 52.9 | 50.3 | 64.1 | 44.0 |
| E07000173 | Gedling | 34.8 | 54.7 | 55.4 | 56.8 | 34.4 |
| E07000174 | Mansfield | 43.0 | 51.6 | 66.8 | 65.0 | 42.2 |
| E07000175 | Newark and Sherwood | 54.9 | 51.1 | 54.4 | 44.4 | 44.4 |
| E07000176 | Rushcliffe | 55.8 | 50.3 | 52.7 | 49.6 | 54.5 |
| E07000177 | Cherwell | 45.5 | 44.1 | 61.0 | 51.8 | 49.9 |
| E07000178 | Oxford | 46.1 | 42.2 | 35.9 | 52.5 | 75.6 |
| E07000179 | South Oxfordshire | 52.9 | 48.4 | 56.2 | 43.4 | 49.6 |
| E07000180 | Vale of White Horse | 51.8 | 52.1 | 50.6 | 37.0 | 55.2 |
| E07000181 | West Oxfordshire | 55.0 | 57.7 | 63.1 | 44.8 | 56.0 |
| E07000187 | Mendip | 46.8 | 43.0 | 44.6 | 58.1 | 62.0 |
| E07000188 | Sedgemoor | 45.2 | 47.5 | 54.2 | 55.6 | 51.0 |
| E07000189 | South Somerset | 49.2 | 54.6 | 58.1 | 57.0 | 48.1 |
| E07000190 | Taunton Deane | 48.1 | 52.3 | 59.9 | 39.1 | 50.8 |
| E07000191 | West Somerset | 56.8 | 79.2 | 61.2 | 32.4 | 49.8 |
| E07000192 | Cannock Chase | 40.7 | 57.4 | 56.4 | 64.5 | 35.9 |
| E07000193 | East Staffordshire | 48.3 | 55.4 | 59.6 | 49.5 | 43.6 |
| E07000194 | Lichfield | 37.8 | 57.6 | 56.6 | 51.4 | 45.3 |
| E07000195 | Newcastle-under-Lyme | 37.6 | 43.8 | 52.0 | 64.8 | 45.9 |
| E07000196 | South Staffordshire | 60.2 | 59.2 | 44.9 | 46.9 | 40.2 |
| E07000197 | Stafford | 47.9 | 53.2 | 51.2 | 41.6 | 42.1 |
| E07000198 | Staffordshire Moorlands | 46.6 | 38.5 | 51.9 | 56.2 | 57.1 |
| E07000199 | Tamworth | 40.6 | 45.9 | 47.0 | 63.1 | 39.7 |
| E07000200 | Babergh | 44.8 | 64.8 | 50.5 | 44.1 | 51.5 |
| E07000201 | Forest Heath | 58.4 | 59.0 | 56.4 | 38.7 | 51.1 |
| E07000202 | Ipswich | 48.4 | 47.9 | 52.9 | 50.2 | 53.0 |
| E07000203 | Mid Suffolk | 60.9 | 60.0 | 63.8 | 36.7 | 43.7 |
| E07000204 | St Edmundsbury | 45.1 | 56.9 | 59.4 | 55.8 | 41.2 |
| E07000205 | Suffolk Coastal | 41.2 | 62.8 | 56.2 | 50.2 | 50.5 |
| E07000206 | Waveney | 32.6 | 57.0 | 53.3 | 57.5 | 50.3 |
| E07000207 | Elmbridge | 76.6 | 41.4 | 60.5 | 27.6 | 51.0 |
| E07000208 | Epsom and Ewell | 65.9 | 49.7 | 50.5 | 47.5 | 45.4 |
| E07000209 | Guildford | 54.6 | 45.4 | 47.7 | 39.4 | 49.0 |
| E07000210 | Mole Valley | 59.1 | 45.8 | 53.3 | 34.7 | 44.6 |
| E07000211 | Reigate and Banstead | 50.0 | 53.3 | 53.4 | 39.5 | 44.0 |
| E07000212 | Runnymede | 66.1 | 49.1 | 53.1 | 42.3 | 46.1 |
| E07000213 | Spelthorne | 63.5 | 59.6 | 57.0 | 36.7 | 48.7 |
| E07000214 | Surrey Heath | 60.5 | 50.5 | 61.1 | 32.7 | 44.8 |
| E07000215 | Tandridge | 50.6 | 41.8 | 66.4 | 33.3 | 53.2 |
| E07000216 | Waverley | 61.1 | 48.9 | 56.2 | 35.3 | 62.0 |
| E07000217 | Woking | 59.9 | 60.5 | 63.5 | 35.7 | 38.6 |
| E07000218 | North Warwickshire | 46.7 | 46.2 | 49.6 | 66.1 | 48.9 |
| E07000219 | Nuneaton and Bedworth | 46.5 | 64.1 | 55.8 | 45.4 | 41.1 |
| E07000220 | Rugby | 48.9 | 38.4 | 45.6 | 55.4 | 45.3 |
| E07000221 | Stratford-on-Avon | 57.6 | 43.2 | 49.1 | 43.4 | 53.6 |
| E07000222 | Warwick | 54.6 | 58.1 | 52.0 | 41.1 | 55.8 |
| E07000223 | Adur | 45.1 | 57.3 | 67.2 | 44.0 | 43.3 |
| E07000224 | Arun | 51.8 | 45.2 | 57.7 | 47.7 | 50.6 |
| E07000225 | Chichester | 63.6 | 52.6 | 60.3 | 31.6 | 66.3 |
| E07000226 | Crawley | 40.4 | 47.8 | 49.6 | 57.4 | 31.1 |
| E07000227 | Horsham | 53.7 | 47.9 | 60.5 | 41.7 | 46.2 |
| E07000228 | Mid Sussex | 45.6 | 48.8 | 60.5 | 44.5 | 45.4 |
| E07000229 | Worthing | 50.1 | 42.7 | 45.3 | 48.0 | 46.9 |
| E07000234 | Bromsgrove | 36.4 | 53.5 | 59.9 | 51.8 | 42.4 |
| E07000235 | Malvern Hills | 36.8 | 45.3 | 52.3 | 53.8 | 62.5 |
| E07000236 | Redditch | 31.3 | 45.4 | 56.9 | 59.9 | 37.0 |
| E07000237 | Worcester | 43.1 | 45.6 | 48.7 | 53.7 | 51.8 |
| E07000238 | Wychavon | 55.8 | 52.7 | 66.6 | 41.4 | 43.3 |
| E07000239 | Wyre Forest | 56.9 | 56.6 | 58.7 | 54.1 | 55.3 |
| E08000001 | Bolton | 50.1 | 43.3 | 44.6 | 47.5 | 48.0 |
| E08000002 | Bury | 48.1 | 45.4 | 48.3 | 50.6 | 44.7 |
| E08000003 | Manchester | 58.6 | 43.8 | 22.4 | 54.5 | 65.3 |
| E08000004 | Oldham | 46.6 | 49.9 | 39.4 | 60.2 | 49.7 |
| E08000005 | Rochdale | 52.3 | 52.6 | 48.5 | 55.9 | 50.4 |
| E08000006 | Salford | 64.2 | 52.2 | 52.4 | 45.3 | 49.3 |
| E08000007 | Stockport | 58.6 | 44.8 | 52.8 | 49.0 | 50.7 |
| E08000008 | Tameside | 56.3 | 53.0 | 45.6 | 57.2 | 51.6 |
| E08000009 | Trafford | 61.4 | 60.3 | 53.3 | 43.6 | 47.6 |
| E08000010 | Wigan | 52.9 | 54.4 | 52.4 | 51.9 | 41.4 |
| E08000011 | Knowsley | 49.3 | 56.6 | 46.8 | 52.7 | 47.0 |
| E08000012 | Liverpool | 45.8 | 43.2 | 29.6 | 62.5 | 61.4 |
| E08000013 | St. Helens | 40.4 | 50.3 | 44.2 | 56.4 | 45.4 |
| E08000014 | Sefton | 52.5 | 47.4 | 44.2 | 47.2 | 47.6 |
| E08000015 | Wirral | 50.4 | 54.8 | 43.6 | 48.8 | 47.9 |
| E08000016 | Barnsley | 50.5 | 47.1 | 51.6 | 59.7 | 38.6 |
| E08000017 | Doncaster | 48.6 | 53.5 | 45.5 | 50.9 | 45.2 |
| E08000018 | Rotherham | 50.6 | 43.0 | 51.8 | 51.6 | 39.0 |
| E08000019 | Sheffield | 50.9 | 40.1 | 36.4 | 62.4 | 49.7 |
| E08000020 | Gateshead | 45.2 | 42.3 | 41.1 | 52.3 | 47.7 |
| E08000021 | Newcastle upon Tyne | 49.3 | 40.5 | 31.2 | 52.7 | 60.4 |
| E08000022 | North Tyneside | 53.9 | 49.0 | 43.5 | 57.4 | 52.6 |
| E08000023 | South Tyneside | 44.4 | 53.3 | 39.0 | 65.2 | 46.8 |
| E08000024 | Sunderland | 42.0 | 71.3 | 51.2 | 44.7 | 53.2 |
| E08000025 | Birmingham | 52.2 | 47.2 | 40.9 | 51.5 | 52.8 |
| E08000026 | Coventry | 40.0 | 43.8 | 38.0 | 57.6 | 50.2 |
| E08000027 | Dudley | 43.2 | 40.3 | 55.1 | 62.6 | 45.4 |
| E08000028 | Sandwell | 43.6 | 31.8 | 41.6 | 74.5 | 50.6 |
| E08000029 | Solihull | 53.6 | 56.2 | 54.3 | 46.1 | 42.2 |
| E08000030 | Walsall | 40.9 | 56.0 | 42.6 | 63.5 | 47.3 |
| E08000031 | Wolverhampton | 39.9 | 43.5 | 45.4 | 60.9 | 41.2 |
| E08000032 | Bradford | 50.6 | 46.7 | 45.7 | 51.2 | 48.8 |
| E08000033 | Calderdale | 39.3 | 49.3 | 43.8 | 59.7 | 54.0 |
| E08000034 | Kirklees | 44.7 | 54.3 | 49.5 | 53.2 | 49.0 |
| E08000035 | Leeds | 56.0 | 43.9 | 38.6 | 52.3 | 53.3 |
| E08000036 | Wakefield | 43.1 | 49.1 | 49.7 | 57.9 | 42.4 |
| E09000001 | City of London | 59.7 | 8.3 | 44.0 | 42.5 | 83.6 |
| E09000002 | Barking and Dagenham | 32.3 | 34.7 | 29.2 | 75.9 | 38.0 |
| E09000003 | Barnet | 63.2 | 43.3 | 35.5 | 52.7 | 65.4 |
| E09000004 | Bexley | 44.9 | 35.4 | 43.5 | 59.2 | 36.0 |
| E09000005 | Brent | 59.0 | 49.0 | 26.8 | 45.4 | 67.4 |
| E09000006 | Bromley | 57.0 | 49.9 | 51.5 | 50.7 | 48.1 |
| E09000007 | Camden | 68.8 | 25.9 | 30.8 | 47.4 | 86.5 |
| E09000008 | Croydon | 54.1 | 41.2 | 42.8 | 49.5 | 53.5 |
| E09000009 | Ealing | 39.5 | 32.8 | 28.5 | 66.0 | 58.9 |
| E09000010 | Enfield | 53.4 | 46.7 | 43.0 | 45.8 | 58.4 |
| E09000011 | Greenwich | 58.8 | 37.2 | 36.1 | 50.7 | 65.0 |
| E09000012 | Hackney | 76.5 | 32.3 | 24.3 | 47.7 | 100.0 |
| E09000013 | Hammersmith and Fulham | 86.7 | 36.5 | 36.0 | 33.5 | 80.2 |
| E09000014 | Haringey | 61.3 | 36.6 | 29.5 | 61.9 | 80.7 |
| E09000015 | Harrow | 51.3 | 37.8 | 31.9 | 56.1 | 54.1 |
| E09000016 | Havering | 46.8 | 48.5 | 52.8 | 48.2 | 42.7 |
| E09000017 | Hillingdon | 46.3 | 48.4 | 49.1 | 58.8 | 47.8 |
| E09000018 | Hounslow | 50.8 | 50.2 | 32.0 | 50.5 | 54.8 |
| E09000019 | Islington | 68.4 | 12.2 | 31.5 | 51.8 | 90.8 |
| E09000020 | Kensington and Chelsea | 75.2 | 9.7 | 29.5 | 52.3 | 88.9 |
| E09000021 | Kingston upon Thames | 61.5 | 39.7 | 45.7 | 48.3 | 59.6 |
| E09000022 | Lambeth | 77.3 | 24.4 | 29.2 | 46.6 | 81.6 |
| E09000023 | Lewisham | 61.3 | 25.7 | 27.3 | 62.7 | 80.5 |
| E09000024 | Merton | 63.5 | 48.1 | 49.6 | 36.2 | 58.6 |
| E09000025 | Newham | 65.5 | 35.5 | 30.8 | 59.0 | 67.9 |
| E09000026 | Redbridge | 52.3 | 37.6 | 35.3 | 50.9 | 54.8 |
| E09000027 | Richmond upon Thames | 79.7 | 36.4 | 44.8 | 39.7 | 72.0 |
| E09000028 | Southwark | 66.7 | 30.0 | 33.5 | 48.4 | 83.5 |
| E09000029 | Sutton | 47.9 | 35.4 | 51.0 | 53.7 | 45.6 |
| E09000030 | Tower Hamlets | 65.5 | 20.7 | 39.9 | 40.8 | 69.7 |
| E09000031 | Waltham Forest | 54.8 | 33.8 | 35.3 | 57.1 | 71.1 |
| E09000032 | Wandsworth | 78.3 | 38.3 | 39.6 | 44.9 | 68.9 |
| E09000033 | Westminster | 69.3 | 20.9 | 37.8 | 34.8 | 83.4 |
| S12000005 | Clackmannanshire | 66.8 | 67.2 | 40.6 | 50.0 | 47.2 |
| S12000006 | Dumfries & Galloway | 32.7 | 65.2 | 45.1 | 51.1 | 54.7 |
| S12000008 | East Ayrshire | 46.0 | 66.4 | 53.3 | 59.3 | 39.2 |
| S12000009 | East Dunbartonshire | 58.1 | 68.9 | 54.6 | 37.5 | 45.4 |
| S12000010 | East Lothian | 54.7 | 53.5 | 49.7 | 55.5 | 51.9 |
| S12000011 | East Renfrewshire | 73.5 | 51.0 | 44.6 | 38.7 | 49.8 |
| S12000013 | Eilean Siar | 45.1 | 64.8 | 61.2 | 23.9 | 60.4 |
| S12000014 | Falkirk | 44.6 | 52.7 | 55.1 | 41.6 | 42.9 |
| S12000015 | Fife | 38.9 | 55.8 | 45.6 | 56.2 | 52.0 |
| S12000017 | Highland | 33.2 | 60.0 | 44.9 | 48.7 | 47.3 |
| S12000018 | Inverclyde | 33.7 | 43.3 | 44.6 | 51.0 | 44.7 |
| S12000019 | Midlothian | 51.3 | 51.5 | 47.1 | 51.5 | 47.7 |
| S12000020 | Moray | 39.5 | 65.1 | 65.5 | 36.0 | 49.5 |
| S12000021 | North Ayrshire | 45.8 | 50.8 | 44.8 | 43.2 | 48.6 |
| S12000023 | Orkney Islands | 35.2 | 51.0 | 58.3 | 4.5 | 64.3 |
| S12000024 | Perth & Kinross | 60.9 | 65.5 | 58.7 | 27.5 | 54.1 |
| S12000026 | Scottish Borders | 46.7 | 57.5 | 45.6 | 49.5 | 49.7 |
| S12000027 | Shetland Islands | 21.2 | 78.3 | 48.2 | 53.2 | 53.9 |
| S12000028 | South Ayrshire | 43.9 | 50.6 | 61.4 | 52.7 | 51.1 |
| S12000029 | South Lanarkshire | 54.4 | 61.6 | 50.5 | 50.3 | 45.4 |
| S12000030 | Stirling | 47.4 | 54.0 | 50.4 | 46.0 | 65.0 |
| S12000033 | Aberdeen City | 41.3 | 38.9 | 33.4 | 56.8 | 47.2 |
| S12000034 | Aberdeenshire | 50.0 | 61.3 | 55.2 | 39.2 | 44.3 |
| S12000035 | Argyll & Bute | 44.3 | 80.0 | 55.1 | 39.9 | 58.4 |
| S12000036 | Edinburgh, City of | 51.1 | 48.9 | 36.9 | 49.4 | 64.9 |
| S12000038 | Renfrewshire | 36.6 | 49.7 | 50.0 | 66.8 | 47.5 |
| S12000039 | West Dunbartonshire | 47.5 | 50.9 | 39.0 | 70.5 | 43.7 |
| S12000040 | West Lothian | 44.2 | 59.6 | 54.0 | 56.1 | 40.1 |
| S12000041 | Angus | 49.2 | 70.3 | 55.4 | 41.4 | 34.6 |
| S12000042 | Dundee City | 53.3 | 50.4 | 34.3 | 58.6 | 55.3 |
| S12000043 | Glasgow City | 54.7 | 39.2 | 27.9 | 63.4 | 61.7 |
| S12000044 | North Lanarkshire | 40.6 | 50.7 | 44.5 | 57.9 | 40.2 |
| W06000001 | Isle of Anglesey | 46.4 | 60.0 | 40.8 | 47.2 | 42.9 |
| W06000002 | Gwynedd | 42.0 | 45.9 | 30.4 | 52.5 | 55.9 |
| W06000003 | Conwy | 45.4 | 55.2 | 47.8 | 37.8 | 56.2 |
| W06000004 | Denbighshire | 54.3 | 70.6 | 54.6 | 47.7 | 56.7 |
| W06000005 | Flintshire | 43.9 | 63.2 | 50.2 | 45.6 | 50.7 |
| W06000006 | Wrexham | 45.6 | 55.1 | 44.1 | 46.7 | 47.8 |
| W06000008 | Ceredigion | 38.2 | 37.5 | 28.6 | 76.1 | 55.6 |
| W06000009 | Pembrokeshire | 47.4 | 50.6 | 46.3 | 52.5 | 55.4 |
| W06000010 | Carmarthenshire | 46.3 | 56.5 | 49.4 | 56.9 | 54.3 |
| W06000011 | Swansea | 53.1 | 51.5 | 37.4 | 47.3 | 55.2 |
| W06000012 | Neath Port Talbot | 63.9 | 53.5 | 39.3 | 55.5 | 45.9 |
| W06000013 | Bridgend | 46.8 | 59.0 | 49.1 | 57.2 | 43.5 |
| W06000014 | The Vale of Glamorgan | 52.1 | 59.1 | 49.8 | 45.2 | 52.9 |
| W06000015 | Cardiff | 57.4 | 49.5 | 34.0 | 47.9 | 56.2 |
| W06000016 | Rhondda Cynon Taf | 44.2 | 47.1 | 37.9 | 62.1 | 49.8 |
| W06000018 | Caerphilly | 38.5 | 53.2 | 58.3 | 50.2 | 38.9 |
| W06000019 | Blaenau Gwent | 32.0 | 46.1 | 35.5 | 77.1 | 40.7 |
| W06000020 | Torfaen | 45.5 | 53.4 | 54.5 | 54.3 | 47.7 |
| W06000021 | Monmouthshire | 44.0 | 58.2 | 52.8 | 43.6 | 47.5 |
| W06000022 | Newport | 41.8 | 42.6 | 42.4 | 67.3 | 48.7 |
| W06000023 | Powys | 38.8 | 46.8 | 46.3 | 64.9 | 62.5 |
| W06000024 | Merthyr Tydfil | 37.5 | 47.7 | 13.6 | 72.3 | 45.4 |

Note. E = Extraversion, A = Agreeableness, C = Conscientiousness, N = Neuroticism, O = Openness.
