# Supplementary material for: Regional Personality Differences in Great Britain
Source: PLoS One. 2015 Mar 24;10(3):e0122245. doi: 10.1371/journal.pone.0122245 (PMC4372610; doi:10.1371/journal.pone.0122245)
Supplement: S1 Survey — (DOCX) [file pone.0122245.s004.docx]

**Big Personality Test**

(http://ssl.bbc.co.uk/labuk/experiments/personality)

**ABOUT YOU – SECTION 1**

- Gender
  - Male
  - Female
- Ethnic group
  - Asian/Asian British - Indian; Pakistani; Bangladeshi
  - Black/black British
  - East/south-east Asian
  - Middle Eastern
  - Mixed race - white and Asian/Asian British
  - Mixed race - white and black/black British
  - Mixed race - other
  - White
  - Other
  - Rather not say
- Main country of residence
  - See HTML list
- Location of main residence growing up
  - Postcode
  - Street name
  - Town or city
  - County
  - I was not born in the UK

**YOUR EDUCATION AND WORK – SECTION 2**

- Highest level of formal schooling:
  - Did not complete GCSE / CSE / O-Levels
  - Completed GCSE / CSE / O-Levels
  - Completed post-16 vocational course
  - A-Levels
  - Undergraduate degree
  - Postgraduate degree
  - I am still in education
- If you are still in education, what is the highest level of education you expect to obtain?
  - Do not expect to obtain GCSE / CSE / O-Levels
  - GCSE / CSE / O-Levels
  - Post-16 vocational course
  - A-Levels
  - Undergraduate degree
  - Postgraduate degree
- Was the majority of your education up to the age of 18 in state or private/fee-paying schools? (If you are under the age of 18, please state the majority up to the present.)
  - State
  - Private
- Occupational status
  - Still at school
  - At university
  - In full time employment
  - Part time employment
  - Self employed
  - Homemaker/full-time parent
  - Unemployed
  - Retired
- Select the category that best describes your occupation.
  - Accounting/finance
  - Administration
  - Business development
  - Consultancy
  - Customer service
  - Education / training
  - Engineering / R and D
  - Executive / senior management
  - Healthcare
  - IT
  - General management
  - Government / military
  - Homemaker
  - Legal
  - Manufacturing/operations
  - Media
  - Medical/science
  - Personnel
  - Professional
  - Purchasing
  - Sales / marketing / advertising
  - Skilled labour
  - Other
- Which of the descriptions best describes the sort of work you do?
  - Professional and technical occupations
  - Higher administrator occupations
  - Clerical occupations
  - Sales occupations
  - Service occupations
  - Skilled worker
  - Semi-skilled worker
  - Unskilled worker
  - Farm worker
  - Other
- What is your total gross annual or weekly household income?
  - Up to £9,999 per annum 199 per week
  - £10,000 to £19,999 per annum (£200 - £389 per week)
  - £20,000 to £29,999 per annum (£390 to 579 per week)
  - £30,000 to £39,999 per annum (£580 to £769 per week)
  - £40,000 to £49,999 per annum (£770 to £969 per week)
  - £50,000 to £74,999 per annum (£970 to £1,449 per week)
  - £75,000 or more per £1,450 or more per week
  - Don't know
  - Rather not say
- Job satisfaction

1 = Disagree strongly

2 = Disagree somewhat

3 = Neither agree nor disagree

4 = Agree somewhat

5 = Agree strongly

- I like my job better than the average person does.
- I am seldom bored with my job.
- Most days I am enthusiastic about my job.
- I am fairly well satisfied with my job.
- I find real enjoyment in my job.

**YOUR PERSONALITY – SECTION 3**

I see myself as someone who:

- - Is talkative
  - Tends to find fault with others
  - Does a thorough job
  - Is depressed, blue
  - Is original, comes up with new ideas
  - Is reserved
  - Is helpful and unselfish with others
  - Can be somewhat careless
  - Is relaxed, handles stress well
  - Is curious about many different things
  - Is full of energy
  - Starts quarrels with others
  - Is a reliable worker
  - Can be tense
  - Is ingenious, a deep thinker
  - Generates a lot of enthusiasm
  - Has a forgiving nature
  - Tends to be disorganized
  - Worries a lot
  - Has an active imagination
  - Tends to be quiet
  - Is generally trusting
  - Tends to be lazy
  - Is emotionally stable, not easily upset
  - Is inventive
  - Has an assertive personality
  - Can be cold and aloof
  - Perseveres until the task is finished
  - Can be moody
  - Values artistic, aesthetic experiences
  - Is sometimes shy, inhibited
  - Is considerate and kind to almost everyone
  - Does things efficiently
  - Remains calm in tense situations
  - Prefers work that is routine
  - Is outgoing, sociable
  - Is sometimes rude to others
  - Makes plans and follows through with them
  - Gets nervous easily
  - Likes to reflect, play with ideas
  - Has few artistic interests
  - Likes to cooperate with others
  - Is easily distracted
  - Is sophisticated in art, music, or literature
  - I see myself as someone who has high self esteem

**YOUR RELATIONSHIPS – SECTION 4**

- Are you currently in an intimate relationship?
  - Yes
  - No
  - Rather not say
- If yes, how would you describe this relationship?
- Married or in a civil partnership
- Living together (not married or in a civil partnership)
- Neither married nor living together
- Not applicable
- How long have you been in this relationship?
  - Less than a year
  - 1 to 5 years
  - 6 to 10 years
  - 11 to 15 years
  - 15 to 20 years
  - Longer than 20 years
  - Not applicable
- Have you been in an intimate relationship before (marriage/civil partnership/living together/not living together) that has since ended?
  - Yes
  - No
  - Rather not say
- If yes, please indicate how the relationship(s) ended:
  - Married/civil partnership ending in divorce
  - More than one marriage/civil partnership ending in divorce
  - Living together ending in separation
  - More than once, living together ending in separation
  - Not living together ending in separation
  - More than once, not living together ending in separation
  - Widowed
  - More than once widowed
  - Rather not say/not applicable
- If yes, what was the duration of your longest intimate relationship?
  - Less than a year
  - 1 to 5 years
  - 6 to 10 years
  - 11 to 15 years
  - 15 to 20 years
  - Longer than 20 years
- Which sex have the majority of your relationships been with?
  - Opposite sex
  - Same sex
  - Both sexes
  - Rather not say
- How many children do you have?
  - None
  - 1
  - 2
  - 3
  - 4
  - 5
  - 6 or more
- If you have children, do some or all of them still live at home?
  - YES
  - NO
  - Not applicable
- Nature of relationships
  - My partner and I share personal information with each other
  - I am strongly attracted to my partner
  - I think my relationship with my partner will last forever
  - I can tell everything to my partner
  - I find my partner sexually attractive
  - I will probably have another love relationship later in my life
  - My partner rarely understands how I feel
  - I tend to feel sexually aroused when my partner is with m
  - I often think of being with other men/women

**YOUR FAMILY – SECTION 5**

Which of the descriptions best describes the sort of work the main breadwinner in your family did when you were 14?

- - Professional and technical occupations
  - Higher administrator occupations
  - Clerical occupations
  - Sales occupations
  - Service occupations
  - Skilled worker
  - Semi-skilled worker
  - Unskilled worker
  - Farm worker
  - Other
  - Retired
  - Don’t know
- Highest level of formal schooling your mother/main female caregiver completed
  - Did not complete GCSE / CSE / O-Levels
  - Completed GCSE / CSE / O-Levels
  - Completed post-16 vocational course
  - A-Levels
  - Undergraduate degree
  - Postgraduate degree
  - Don’t know
  - Not applicable
- Highest level of formal schooling your father/main male caregiver completed
  - Did not complete GCSE / CSE / O-Levels
  - Completed GCSE / CSE / O-Levels
  - Completed post-16 vocational course
  - A-Levels
  - Undergraduate degree
  - Postgraduate degree
  - Don’t know
  - Not applicable
- Number of siblings
  - 0
  - 1
  - 2
  - 3
  - 4
  - 5
  - 6 or more
- Birth order
  - First born
  - Second born
  - Third born
  - Fourth born
  - Fifth born
  - Sixth or subsequent born

**YOUR CHILDHOOD – SECTION 6** [SKIPPABLE]

- For what proportion of your childhood were you brought up in a single-parent household?:
  - None
  - Some
  - Most
  - All
- Were you born prematurely? (Before 37 weeks)
  - Yes
  - No
  - Rather not say / do not know
- If so, did you experience long-term hospitalisation as a baby?
  - Yes
  - No
  - Rather not say
  - Not applicable
- Childhood traumatic events
  - Did you experience a death of a parent or other close family member?
    - - Yes
      - No
      - Rather not say
- How old were you?
  - - 0-2
    - 3-5
    - 6-8
    - 9-11
    - 12-14
    - 15-17
    - Not applicable
  - How traumatic was this?
    - 1
    - 2
    - 3
    - 4
    - 5
    - 6
    - 7
    - Not applicable
  - Was there a major upheaval between your parents (such as divorce, separation)?
    - - Yes
      - No
      - Rather not say
- How old were you?
  - - 0-2
    - 3-5
    - 6-8
    - 9-11
    - 12-14
    - 15-17
    - Not applicable
  - How traumatic was this?
    - 1
    - 2
    - 3
    - 4
    - 5
    - 6
    - 7
    - Not applicable
  - Did you have a traumatic sexual experience (raped, molested, etc.)?
    - - Yes
      - No
      - Rather not say
- How old were you?
  - - 0-2
    - 3-5
    - 6-8
    - 9-11
    - 12-14
    - 15-17
    - Not applicable
  - How traumatic was this?
    - 1
    - 2
    - 3
    - 4
    - 5
    - 6
    - 7
    - Not applicable
  - Were you the victim of violence (child abuse, mugged or assaulted -other than sexual)?
    - - Yes
      - No
      - Rather not say
- How old were you?
  - - 0-2
    - 3-5
    - 6-8
    - 9-11
    - 12-14
    - 15-17
    - Not applicable
  - How traumatic was this?
    - 1
    - 2
    - 3
    - 4
    - 5
    - 6
    - 7
    - Not applicable

**YOUR HEALTH & LIFESTYLE – SECTION 7**

Health

1. In general, would you say your health is:

Excellent

Very good

Good

Fair

Poor

2. During the past 4 weeks, have you accomplished less with your work or other daily activities than you would like as a result of your physical health?

Yes

No

3. During the past 4 weeks, how much did pain interfere with your normal work (including both work outside the home and housework)?

Not at all

A little bit

Moderately

Quite a bit

Extremely

4. How much bodily pain have you had during the past 4 weeks?

None

Very mild

Mild

Moderate

Severe

Very Severe

5. How much of the time during the past 4 weeks have you had a lot of energy?

All of the time

Most of the time

A good bit of the time

Some of the time

A little bit of the time

None of the time

6. During the past 4 weeks, to what extent have your physical health or emotional problems interfered with your normal social activities with family, friends, neighbours or groups?

Not at all

Slightly

Moderately

Quite a bit

Extremely

7. How much of the time during the past 4 weeks have you felt so down in the dumps that nothing could cheer you up?

All of the time

Most of the time

A good bit of the time

Some of the time

A little of the time

None of the time

8. During the past 4 weeks have your accomplished less than you would like with your work or other daily activities as a result of any emotional problem (such as feeling depressed or anxious)?

Yes

No

- Have you ever smoked cigarettes daily, that is, at least one cigarette every day for 30 days?
  - Yes
  - No

- Height [imperial, metric]
- Weight [imperial st/lbs and lbs, metric]
- During the past 30 days, on average how many cigarettes did you smoke per day?
  - None
  - Less than 1 cigarette per day
  - 1 cigarette per day
  - 2 to 5 cigarettes per day
  - 6 to 10 cigarettes per day
  - 11 to 20 cigarettes per day
  - More than 20 cigarettes per day
- During the past 30 days, on how many days did you have at least one drink of alcohol?
  - 0 days
  - 1 or 2 days
  - 3 to 5 days
  - 6 to 9 days
  - 10 to 19 days
  - 20 to 29 days
  - All 30 days
- During the past 30 days, on how many days did you have 5 or more drinks of alcohol in a row, that is, within a couple of hours?
  - 0 days
  - 1 day
  - 2 days
  - 3 to 5 days
  - 6 to 9 days
  - 10 to 19 days
  - 20 or more days
- During your life, have you ever used ‘recreational’ drugs? (A drug, such as marijuana, used non-medically for personal enjoyment.)
  - 0 times
  - 1 or 2 times
  - 3 to 9 times
  - 10 to 19 times
  - 20 to 39 times
  - 40 to 99 times
  - 100 or more times
- Have you ever had sexual intercourse?
- Yes
- No
- Rather not say
- If yes, how old were you when you had sexual intercourse for the first time?
- Under 16 years old
- 16 years old or older
- Rather not say/not applicable
- If yes, during your life, with how many people have you had sexual intercourse?
  - 1 to 5
  - 6 to 10
  - 11 to 15
  - 15 to 25
  - 25 to 50
  - More than 50
  - Rather not say / not applicable
- During the last 30 days, on how many days did you exercise for a total of at least 60 minutes per day? (Include any kind of physical activity that increased your heart rate and made you breathe hard some of the time.)
  - 0 days
  - 1 or 2 days
  - 3 to 5 days
  - 6 to 9 days
  - 10 to 19 days
  - 20 to 29 days
  - All 30 days
- During the last 30 days, how often did you have trouble sleeping?
  - 0 days
  - 1 or 2 days
  - 3 to 5 days
  - 6 to 9 days
  - 10 to 19 days
  - 20 to 29 days
  - All 30 days
  - Do not know
- How often have you been hospitalised (in your life) through accident or injury?
  - None
  - Once
  - Twice
  - 3 times
  - 4 times
  - 5 times
  - 6 or more
- In the last year, have you worked more than 40 hours per week most weeks?
  - Yes
  - No
  - Rather not say
- In general, how stressful do you find your daily life?
  - Not at all
  - A little
  - Moderately
  - Very

**YOUR ASPIRATIONS AND HAPPINESS – SECTION 8**

- Goals
  - 1. Unimportant
    2. Fairly unimportant
    3. Neither important nor unimportant
    4. Quite important
    5. Very important
- Having fun
- Being an accomplished musician
- Having a satisfying marriage/relationship
- Having a high standard of living and wealth
- Owning my own business
- Being influential in public affairs
- Participating in religious activities
- Producing good artistic work
- Writing good fiction and prose
- Having new and different experiences
- Having an exciting lifestyle
- Helping others in need
- Having an influential and prestigious occupation
- Having a high-status career
- Taking part in volunteer community and public service
- Working to promote the welfare of others
- Becoming accomplished in one of the performing arts
- Devoting attention to my spiritual life
- Becoming a community leader
- Making my parents proud
- Having children
- Having harmonious relationships with my parents and my siblings

Life satisfaction

Below are five statements with which you may agree or disagree.

- - - - Strongly agree
      - Agree
      - Slightly agree
      - Neither agree nor disagree
      - Slightly disagree
      - Disagree
      - Strongly disagree
  - In most ways, my life is close to my ideal.
  - The conditions of my life are excellent.
  - I am satisfied with my life.
  - So far, I have got the important things I want in life.
  - If I could live my life over, I would change almost nothing.

Would you like to receive email updates about the Big Personality Test?

- **Yes**
- **No**
